# Eco-Friendly Synthesis of AuPd Bimetallic Nanoparticles Using *Alpinia zerumbet* for Efficient Reduction of Nitro Compounds

**DOI:** 10.1021/acsomega.5c09011

**Published:** 2026-03-18

**Authors:** Ana Paula Nazar de Souza, Gabriel Francisco Souza da Silva, Evelyn C. S. Santos, Jefferson S. de Gois, Cesar Augusto D. Mendoza, Suellen Dayenn Tozetti de Barros, Marcelo E. H. Maia da Costa, Marcelo Augusto Vieira de Souza, Luiz Fernando B. Malta, Nakédia M. F. Carvalho, Jaqueline D. Senra

**Affiliations:** † Chemistry Institute, Rio de Janeiro State University, Rua São Francisco Xavier 524 Maracanã, Rio de Janeiro 20550-900, Rio de Janeiro, Brazil; ‡ 28125 Rio de Janeiro Federal University, Av. Athos da Silveira Ramos 149, Bloco A, Cidade Universitária,Rio de Janeiro 21941-909, Rio de Janeiro, Brazil; § Centro Brasileiro de Pesquisas Físicas, Urca, Rio de Janeiro 22290-180, Rio de Janeiro, Brazil; ∥ Department of Electric Engineering, Rio de Janeiro State University, Rua São Francisco Xavier 524 Maracanã, Rio de Janeiro 20550-900, Rio de Janeiro, Brazil; ⊥ Department of Physics, Pontifical Catholic University of Rio de Janeiro, Rua Marques de São Vicente 22451-900, Rio de Janeiro, Brazil

## Abstract

The development of sustainable nanocatalysts is an important challenge in modern chemistry, with the aim of replacing conventional synthesis routes that employ toxic reagents and harsh conditions. In this work, we report the eco-friendly synthesis of AuPd bimetallic nanoparticles, using *Alpinia zerumbet* extract as a natural reducing and stabilizing agent. 2-Hydroxypropyl-β-cyclodextrin was also used as an additive for the reduction of Pd­(II). A detailed characterization confirmed the successful formation of AuPd NPs with a heterogeneous size distribution and the coexistence of small nanoparticles (2–3 nm) along with larger aggregates. The catalytic activity was evaluated in the reduction of nitroaromatic compounds (nitrobenzene, 3-nitroaniline, and 4-nitrophenol) in water, using NaBH_4_ under mild heating. A maximum turnover frequency (TOF) of 393 h^–1^ was observed for the reduction of nitrobenzene. The AuPd NPs exhibited enhanced catalytic activity, suggesting a remarkable synergy between the two metals associated with the presence of alloy effects. Overall, these findings highlight the potential of *A. zerumbet*-mediated AuPd nanoalloys as sustainable and highly efficient nanocatalysts for the detoxification of nitroaromatic pollutants and the production of value-added amines.

## Graphical abstract

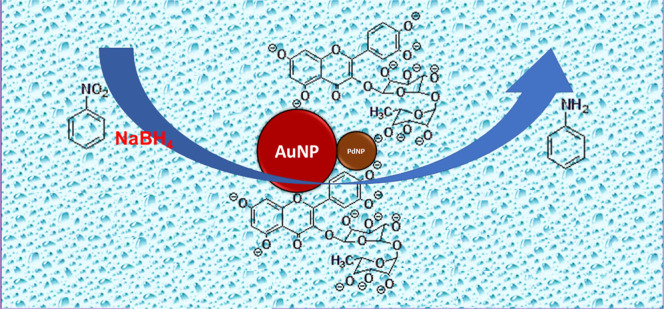

## 1 Introduction

The catalytic hydrogenation of nitro compounds to the corresponding amino derivatives offers a plethora of opportunities toward a variety of fine and specialty chemicals. In addition, some nitro compounds such as nitroarenes are carcinogenic contaminants and toxic biorefractories to living organisms.^1–7^ Thus, the reduction reaction represents a convenient method for transforming them into environmentally less hazardous products besides being a strategic reaction for the synthesis of high-valued amine derivatives.^7–14^

The selective catalytic hydrogenation can be mostly performed by metallic systems, especially those containing noble metal components. Particularly, Ag-, Au-, and bimetallic systems have emerged as some of the most effective heterogeneous catalysts for the reduction of dyes and nitroaromatics.^15^ For example, silver nanoparticles supported on polymeric spheres exhibited strong catalytic activity and excellent stability for dye reduction.^16^ Similarly, zeolite-confined Ag nanoparticles have been shown to achieve ultrafast reduction of 4-nitrophenol and methylene blue due to the ultrasmall Ag size.^17^ Beyond Ag systems, Au-based nanocatalysts integrated with g-C_3_N_4_ nanotubes and CeO_2_–Au/SnO_2_ heterostructures demonstrated remarkable synergistic effects, enabling exceptionally high rate constants for the nitroaromatic reduction.^18^ Bimetallic-based nanosystems often display enhanced catalytic activity and selectivity that can be related to their special ensemble and ligand effects.^19^ Among bimetallic and alloy catalysts, AuPd has received a great deal of attention because of its superior activity in a number of catalytic reactions.^20–22^ In particular, the unique ability of Au to gain s and p electrons, and lose d electrons, promotes Pd d-band perturbation and the consequent weakening of the binding strength.^20^ Thus, Pd catalytic sites are frequently found to be activated within AuPd NPs when compared to pure Pd, mostly due to a decrease in self-poisoning by reactants/products.^23^ Recent studies have demonstrated that bimetallic nanostructures, such as AuPd nanoalloys, can exhibit remarkable synergistic effects in the catalytic reduction of organic pollutants. For instance, Bingwa et al. reported that dendrimer-encapsulated AuPd nanoparticles supported on mesoporous metal oxides display significantly enhanced catalytic performance toward 4-nitrophenol reduction, primarily due to the highly efficient support-mediated electron transfer and the excellent dispersion of the bimetallic nanoparticles within the dendrimer matrix.^24^

However, the chemical and physical synthesis of these nanoparticles generally involves components and processes that are not environmentally friendly, employing strong acids and bases such as HCl, H_2_SO_4_, and NaOH, operating at high temperatures for extended periods, in addition to using reducing agents and stabilizers that generate toxic byproducts.^25–27^

Green synthesis of nanoparticles, employing phytochemicals such as terpenoids, glycosides (e.g., cyclodextrins), alkaloids, and phenolic compounds (e.g., flavonoids, coumarins, ubiquinones, etc.) can mitigate the ecological impact caused by the catalyst preparation due to the milder conditions applied. These biomolecules can promote the synthesis of metal nanoparticles; however, the advantage lies in the fact that the reducing and stabilizing agents are natural substances, preventing the generation of toxic byproducts and excessive energy consumption required to the conventional synthesis procedures.^25,26^

*Alpinia zerumbet* (Pers.) BL Burtt & RM Sm. is a plant native to Western Asia and traditionally used in Brazil for its medicinal properties. Its leaves and fruits are used in traditional medicine and to obtain essential oils with medicinal properties. Although the flower may also have pharmacological potential, the most commonly documented use is for the leaves, which exhibit antimicrobial, antioxidant, and anti-inflammatory activities, including antimicrobial, anti-inflammatory, antioxidant, antihypertensive, diuretic, and analgesic activities.^27^ These therapeutic effects are attributed to the phytochemicals present in the plant, such as chalcones (pinocembrin and cardamonin), flavonoids (kaempferol and pinostrobin), monoterpene esters, diarylheptanoids, and neolignans, among others. These phenolic compounds also exhibit the ability to reduce and stabilize metal ions, a property that has been little explored in recent years.^28–30^ Li et al. reported the use of *A. zerumbet* leaves in the synthesis of silver nanowires (AgNWs) without the need of chemical stabilizers such as polyvinylpyrrolidone (PVP), where the phytochemicals extracted from the leaves acted as both a reducing agent and a template during the hydrothermal synthesis process.^31^

Cyclodextrins (CDs) are oligosaccharides with a hydrophilic outer surface and a hydrophobic inner cavity, which enables them to form inclusion complexes that can reduce metal precursors and stabilize metallic nanoparticles.^32,33^ Furthermore, the use of CDs in chemical reactions can lead to the formation of dynamic supramolecular aggregates that can be energetically favorable due to the strong interactions occurring within the confined space. Their solubility in aqueous media also allows organic reactions to be carried out in water without the need for flammable or carcinogenic organic solvents.^34,35^

Recently, we found that AuPd bimetallic systems prepared by the citrate–CD method did not exhibit metallic alloy behavior.^3^ Despite this, the photocatalytic activation associated with localized surface plasmon resonance (LSPR) demonstrated significant synergy between Au and Pd, with Au sites acting as antennas, absorbing light energy and transferring it to the Pd catalyzed reactions. The use of specific stabilizers and reducing agents for Au and Pd is essential because nanoparticles of these metals require morphological control during the synthesis, particularly to obtain nanoalloys where the synergistic effect enhances the catalytic activity of each metal, enabling reactions under mild conditions with high yields.^3,34,36–39^

Therefore, the present work focuses on the investigation of *A. zerumbet* toward the formation of AuPd NPs along with the use of the bimetallic NPs in the rapid reduction of nitroaromatic compounds in aqueous medium. Furthermore, we propose the synthesis of AuPd nanoalloys by replacing sodium citrate, commonly used as a reducing and stabilizing agent for Au NPs, for *A. zerumbet* extract and the use of 2-hydroxypropyl-β-cyclodextrin (β-HPCD) as an additional reducing and stabilizing agent for Pd NPs. This work was structured into two main stages: (i) the synthesis and characterization of AuPd nanoalloys and (ii) their subsequent application in the model reduction reaction of nitroaromatic compounds such as nitrobenzene, 4-nitrophenol (4-NP), and 3-nitroaniline (3-NA).

## 2 Experimental Section

### 2.1 Materials

Sodium tetrachloroaurate­(III), (NaAuCl_4_·2H_2_O), sodium tetrachloropalladate­(II) (Na_2_PdCl_4_), 2-hydroxypropyl-β-cyclodextrin (β-HPCD), sodium borohydride (NaBH_4_), nitrobenzene (NB), 4-nitrophenol (4-NP), 3-nitroaniline (3-NA), NaCl, anhydrous Na_2_SO_4_, and Folin–Ciocalteu phenol reagent (2 mol L^–1^) were purchased from Sigma-Aldrich. Ethyl acetate and calcium carbonate (99%) were purchased from NEON and Merck, respectively. All aqueous solutions were prepared using distilled water.

### 2.2 Synthesis

#### 2.2.1 Hydroalcoholic Extract of *A. zerumbet*

The extract was freshly prepared by infusing 5 g of the leaves of *A. zerumbet* in 40 mL of distilled water (125 g·L^–1^) at 80 °C for 1 h. The infusion was then filtered under reduced pressure and by a nonsterile 0.45 μm Millipore cellulose nitrate membrane and subsequently cooled to room temperature (25 °C).

#### 2.2.2 Green Synthesis of AuPd Nanoparticles

The green synthesis of AuPd NPs was carried out via a two-step procedure, starting with the preparation of gold nanoparticles (Au NPs). In the first step, 3.1 mL of a 4 mM NaAuCl_4_·2H_2_O solution (12.5 μmol of Au^3+^) was diluted to a final volume of 17.2 mL, yielding a solution with a final concentration of approximately 0.7 mM Au^3+^. The solution was heated under magnetic stirring in an oil bath. Once reflux was established, 6 mL of the hydroalcoholic extract of *A. zerumbet* was added dropwise, and the reaction was maintained under reflux for an additional 30 min, leading to the reduction of Au^3+^ ions and the formation of Au NPs.

In the second step, 1.3 mL of a 4 mM Na_2_PdCl_4_ solution (5 μmol of Pd^2+^) was introduced into 9.6 mL of the previously prepared Au NP suspension, resulting in a final Pd^2+^ concentration of approximately 0.5 mM. The mixture was heated at 80 °C under magnetic stirring in an oil bath. Subsequently, 345 mg of 2-hydroxypropyl-β-cyclodextrin (β-HPCD, 0.3 mmol) was added, establishing a Au:Pd:β-HPCD molar ratio of approximately 0.9:1:60. The synthesis continued at 80 °C for 30 min. A fraction of the resulting AuPd suspension was lyophilized for further characterization by X-ray diffraction (XRD).

### 2.3 Characterization

The *A. zerumbet* extract was first evaluated using the Folin–Ciocalteu assay to estimate the total phenolic content, followed by complementary qualitative tests to identify the presence of major phytochemical classes. High-performance liquid chromatography with diode-array detection (HPLC-DAD) was then employed to determine the specific phenolic profile, while cyclic voltammetry was performed to assess the extract’s redox properties and antioxidant potential. All equipment, reagents, and methods used in the extract characterization tests are described in the Supporting Information.

Structural characterization of the bimetallic nanoparticles was conducted by XRD on a Bruker D8 Advance diffractometer operated at 40 kV and 40 mA, within a 2θ range of 10–60° and a step size of 0.02°. Optical properties were evaluated using UV–vis spectroscopy with an Agilent 8453 diode-array spectrophotometer (USA, UERJ). Scanning electron microscopy (SEM) images were obtained with a JEOL JSM-6701F field-emission scanning electron microscope equipped with an energy-dispersive X-ray spectroscopy (EDS) system, or alternatively with a JEOL 7100FT microscope. Transmission electron microscopy (TEM) was carried out using a JEOL 2100F instrument (LABNANO/CBPF) operated at 200 kV. TEM specimens were prepared by drop-casting the colloidal suspensions onto carbon-coated copper grids (400 mesh). High-resolution TEM (HRTEM) images were acquired with a 16-megapixel CCD camera (OneView Orius), and additional scanning TEM (STEM) analyses were conducted. Elemental composition and spatial distribution were examined by EDS with a probe size of 1 nm. Hydrodynamic size distribution and colloidal stability were assessed by dynamic light scattering (DLS) on a Malvern Zetasizer Nano S90 (UERJ), and zeta potential measurements were performed with a Malvern Zetasizer PRO (CBPF). FTIR analyses were carried out in a PerkinElmer Frontier Single & Dual Ranger (USA), with the samples prepared as KBr pellets. X-ray photoelectron spectroscopy (XPS) experiments were performed using a SPECS Phoibos 150 spectrometer in a surface analysis chamber under ultrahigh vacuum (pressure of approximately 10^–8^ Pa). The measurements were acquired with an Al Kα nonmonochromatic X-ray source (1486.7 eV). The C 1s peak at 284.8 eV was used as an internal standard to compensate for effects related to charge shift. The spectra were deconvoluted in CasaXPS software (Fairley et al., 2021), using a pseudo Voigt function with Gaussian–Lorentzian, 40% Lorentzian, and a Shirley-type background.

### 2.4 Reduction of Nitro Compounds

The catalytic activity of the AuPd nanoalloys was investigated in nitroaromatic reduction reactions. Nitrobenzene (NB) was selected as the benchmark substrate to optimize the reaction conditions. The reactions were performed in triplicate under mild heating at 60 °C, maintaining a molar ratio of 2.5 NaBH_4_:1 NB and employing an approximately 1.0 mol % AuPd catalyst relative to the substrate.

Representative experimental procedure: In a round-bottom flask, a mixture of nitrobenzene (2 μmol), NaBH_4_ (2 μmol) and 360 μL (2 × 10^–3^ μmol) of the bimetallic dispersion was adjusted to a final volume of the 5 mL with water. The mixture was magnetically stirred at 60 °C for 15 min under irradiation or in the dark. After completion of the reaction, the reaction medium was extracted with ethyl acetate/brine (1:1, v/v; 3 × 5 mL), dried under anhydrous Na_2_SO_4_, filtered, and evaporated under reduced pressure to afford the phenylamine.

The experiments carried out under light were performed with a 150 W halogen lamp (KIAN).

The quantification of the products was performed by gas chromatography (GC) on an Agilent Technologies 7890B system equipped with a flame ionization detector. The conversion, yield, and selectivity were calculated, as detailed in the Supporting Information. Proton nuclear magnetic resonance (^1^H NMR) spectra were acquired on a Bruker AV-500 spectrometer at 25 °C.

## 3 Results and Discussion

### 3.1 Characterization of the Hydroalcoholic Extract of *A. zerumbet*

The polyphenolic compounds play a dual role in the green synthesis of metallic nanoparticles: as reducing agents, while their hydroxyl and aromatic groups act as stabilizing and capping agents, preventing aggregation. A variety of structures, including kavalactones, chalcones, flavonoids, diterpenoids, and sesquiterpenoids, have been isolated from *A. zerumbet*, as illustrated in Figure S1. This biomolecule-mediated synthesis not only eliminates the need for hazardous reducing agents but can also confer surface functionalities that improve catalytic efficiency in redox reactions, making *A. zerumbet* extract promising for the environmentally friendly production of nanocatalysts.

The extract of *A. zerumbet* exhibited a total phenolic content of 2.41 mg g^–1^, with the qualitative analyses indicating the presence of tannins, phenols, and flavonoids. HPLC-DAD analysis identified the presence of the flavonoid rutin, gallic acid, and caffeic acid as constituents of the aqueous extract of *A. zerumbet*. The chromatogram provided in Figure S2 further indicates the presence of multiple organic compounds within the extract composition.

Cyclic voltammetry was employed to investigate the redox properties of the *A. zerumbet* extract. The voltammogram (Figure S3) revealed irreversible anodic and cathodic processes, with an oxidation peak *E*_pa_ = +500 mV vs NHE (NHE = normal hydrogen electrode) and a reduction peak *E*_pc_ = +410 mV vs NHE, confirming the presence of electroactive species. The redox process is typical of rutin which is the major polyphenol determined by HPLC (Figure S2).^40^ These redox-active compounds exhibit potentials substantially lower than the standard reduction potentials^41^ of Au^3+^/Au (*E*° = 1.498 V vs NHE) and Pd^2+^/Pd (*E*° = 0.987 V vs NHE), suggesting that the extract can act as an effective reducing agent for these metal ions under mild conditions. Although kinetic and complexation factors may modulate the reaction pathway, the observed electrochemical profile supports an electron-donating capability compatible with the nucleation of Au metallic nanoparticles. In addition, the polyphenolic components likely serve as capping agents, stabilizing the growing nanostructures and preventing aggregation, thereby enhancing colloidal stability.^39,40^ Taken together, these findings support the hypothesis that the electroactive compounds in *A. zerumbet* extract not only drive the reduction of metal precursors but also promote the formation of stable, catalytically active nanoparticles, positioning this extract as a sustainable alternative for green nanomaterial synthesis.^42^ However, from the viewpoint of the individual Pd­(II) reduction reaction, some qualitative controls indicated the characteristic coloration of the Pd(0) formation but a lack of sufficient stability over an extended time. Thus, the addition of β-HPCD was chosen as a known additive for the promotion of Pd NPs stability.^43^

### 3.2 Characterization of the Nanomaterials

Figure 1a,b displays the powder XRD patterns of lyophilized AuPd NPs and β-HPCD, with a magnified view of the main reflections. The AuPd diffractogram shows an amorphous pattern associated with β-HPCD, together with well-defined peaks corresponding to metallic gold. The diffraction peaks at 2θ = 38.4° and 44.6° are indexed as the (111) and (200) planes of Au, respectively, in agreement with the standard JCPDS card no. 04-0784, which corresponds to face-centered cubic (fcc) gold.^44^ The absence of a distinct Pd (111) reflection, typically expected near 40.1° (JCPDS no. 05-0681, fcc Pd), suggests the confinement of these NPs in small crystallite sizes which are below the detection limit of the XRD instrument.^45^ The dominance of the Au (111) peak further indicates preferential orientation along this plane, which is frequently observed in metallic nanoparticles due to its thermodynamic stability.^34,45–47^

**Figure 1:**
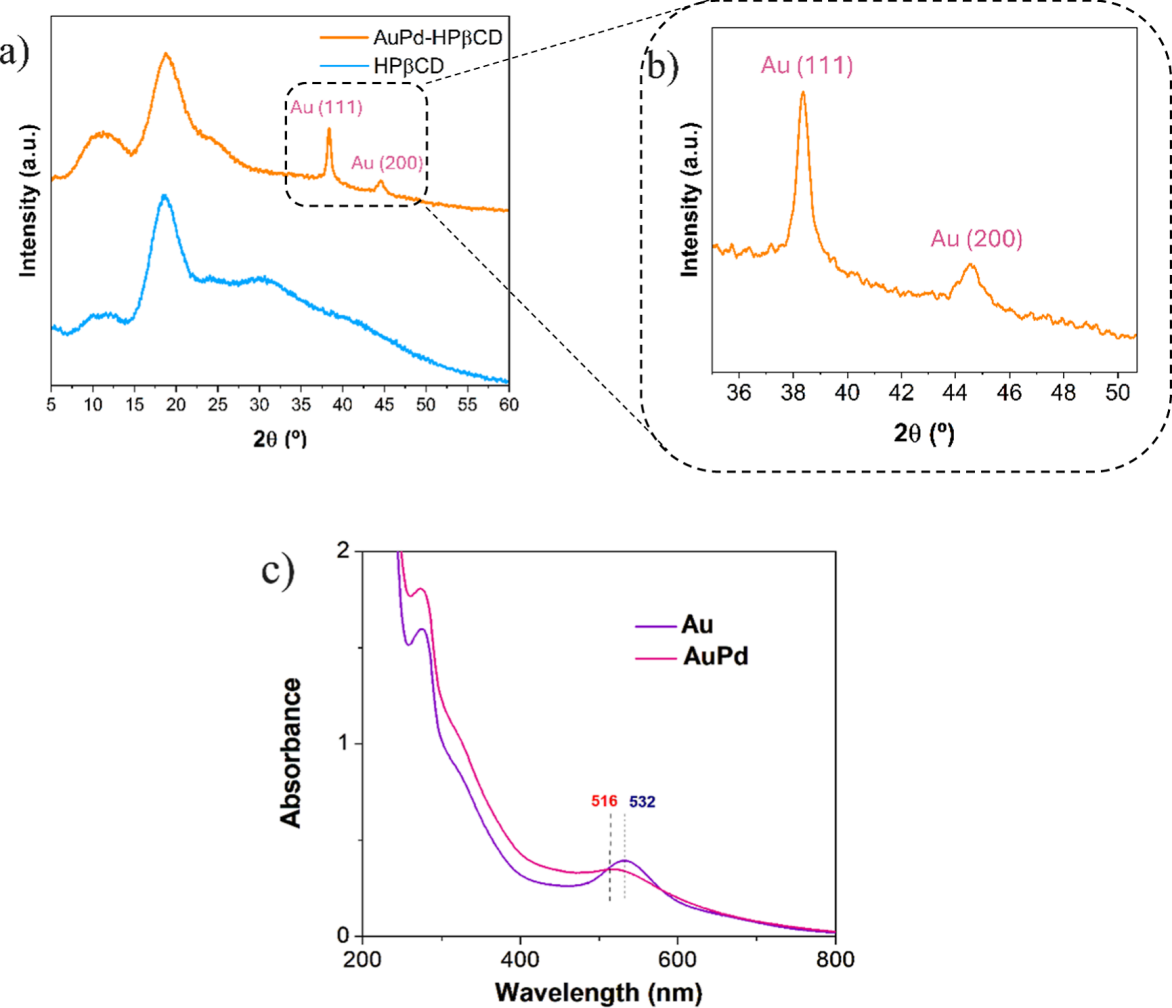
(a) Powder X-ray diffractograms for AuPd NPs; (b) zoomed-in region related to the (111) and (200) reflections of Au; and (c) UV–vis spectra for Au and AuPd NPs dispersion.

Additionally, UV–vis absorption spectroscopy was performed on the suspensions of the Au NPs and AuPd NPs (Figure 1c).^7,34,48^ The absorption band observed at approximately 532 nm corresponds to the LSPR of Au NPs, which originates from the collective oscillation of the conduction band electrons under electromagnetic irradiation.^49^ The slight blue shift of this band from 532 to 516 nm suggests alloy formation and possible size reduction, as previously reported for bimetallic systems.^8^ Furthermore, a distinct absorption band at approximately 270 nm was observed, which is attributed to phytochemicals present in the *A. zerumbet* extract, as shown in the UV–vis spectrum of the extract (Figure S4). This region of the spectrum is commonly associated with aromatic compounds containing conjugated systems, such as flavonoids (kaempferol, quercetin derivatives, flavanones) and phenolic acids (gallic, caffeic, and ferulic acids), which exhibit π → π* electronic transitions in the benzoyl system of the aromatic rings.^50,51^ These findings are consistent with previous reviews reporting that polyphenolic compounds are the main contributors to UV absorption in the 240–280 nm range in plant-based extracts employed for green synthesis of metallic nanoparticles.

FT-IR spectroscopy of AuPd NPs (Figure S5) indicated the presence of functional groups mainly related to the phenolics and β-HPCD. For instance, (i) the intermolecular H-bonded O–H stretching vibrations of alcohols in 3410 cm^–1^, (ii) the H–O–H bending vibration of associated water at 1645 cm^–1^, (iii) the C=C stretching vibrational mode related to the aromatic rings between 1460–1510 cm^–1^, (iv) the CH_2_ stretching mode at 2930 cm^–1^, and (v) the C–O bond stretching (primary and secondary OH groups) at 1079 and 1032 cm^–1^.^43,52^ In addition, the low intensity band observed at 1730 cm^–1^ can be mostly associated with the C=O stretching resultant from the partial HPCD oxidation during the Pd­(II)-to-Pd(0) reduction process.^43^

The SEM-EDX analyses of the AuPd NPs are shown in the Supporting Information. The low-magnification SEM images show an undefined morphology, with the corresponding EDS mapping evidencing a low Au dispersion on the surface. Indeed, the absence of Pd site detection is consistent with the data obtained by XRD (Figure S6). To better investigate the microstructural properties of AuPd NPs, we performed TEM analyses of both Au and AuPd nanoparticles. As observed in Figure 2a, the Au NPs exhibit predominantly spherical and triangular morphologies with sizes ranging from 20 to 60 nm. The AuPd bimetallic NPs (Figure 2b,c) clearly display a bimodal size distribution, with the smaller nanoparticles showing an average size of approximately 2.6 ± 0.4 nm (Figure 2e). According to energy-dispersive X-ray spectroscopy (EDS) analyses (Figure 2g–i), the larger nanoparticles contain both Au and Pd, suggesting the formation of AuPd nanoalloys, with the smaller ones predominantly constituted of Pd. Selected bimetallic AuPd nanoparticles were also analyzed by electron diffraction (Figure 2f), confirming their crystalline nature. In addition, when a fast Fourier transform was performed on the HRTEM image, the distance of 1.99 Å could be measured (Figure 2d), which should correspond to the {200} plane of the face-centered cubic (fcc) structure of palladium. Therefore, according to HRTEM images, it seems that palladium is mainly localized in discrete regions that exhibit a narrow dispersion throughout the bimetallic NPs sample. We also calculated the crystallite size of the AuPd NPs by the Au diffraction peak at 38.4° using the Scherrer equation.^53,54^ In this case, we obtained the value of 16 nm. A comparison with the AuPd particle size range observed by TEM (20–60 nm) suggests that the bimetallic NPs may be composed of 1–3 crystallites. It is worth noting that the smaller NPs observed in Figure 2 mainly composed of Pd—have a mean size of 2.6 nm, which does not allow diffracting enough light in order to be detected by XRD. XPS analyses of AuPd NPs were also performed, and the results indicated the presence of Pd in the form of both Pd(0) and PdO, indicated in the Pd 3d edge by spin–orbit coupling, with binding energies corresponding to the 3d_5_/_2_ sublevel at 335.4 and 336.3 eV, respectively (Figures S7).^55,56^ However, the strong loading effect in the Au spectrum hampered further conclusions.

**Figure 2:**
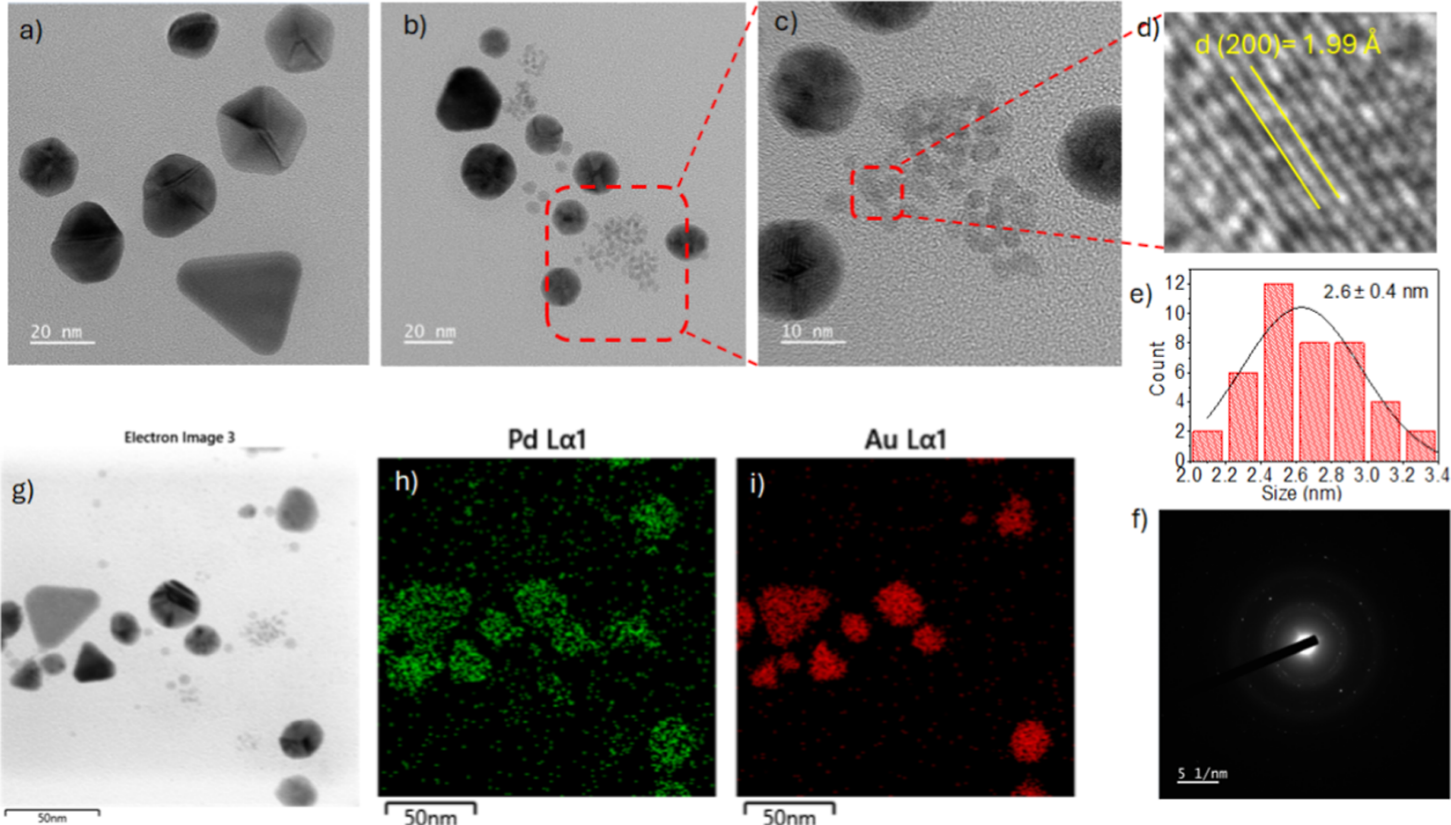
Bright-field representative TEM images of Au NPs (a) and AuPd NPs (molar ratio of Au:Pd:β-HPCD, 0.9:1:60) with the chosen area marked in the red dashed box for high resolution (b). HRTEM of AuPd NPs showing the chosen area marked in the red dashed box for crystal d-spacing measurements (c, d). Histogram with the particle size distribution of the smallest AuPd NPs (e), and its corresponding SAED image (f). Low-magnification TEM image of AuPd NPs (g) and EDS elemental mapping showing the Pd (h) and Au (i) distributions, represented as green and red dots, respectively.

It is worth noting that in contrast to the previously reported AuPd NPs prepared via the citrate–cyclodextrin method, which mainly consisted of a physical mixture of approximately 20 nm AuNPs and ultrasmall Pd NPs (2–3 nm) stabilized by β-HPCD without significant alloy formation, the present system indicates a strong evidence toward the formation of AuPd nanoalloys using *A. zerumbet* extract as a natural reducing and stabilizing agent for gold.

It is well established that the segregation of bimetallic AuPd alloy-like NPs is thermodynamically driven with some possible analytical solutions. Apart from the fact that AuPd can form a miscible metal alloy, the factors leading to the degree of segregation along with the formation of a secondary Pd phase (or enriched phase) will depend on the morphological (e.g., the relative size of the surface compared to the subsurface) and AuPd ratio. In fact, these nonlinear effects can be caused by the changes in the Fermi level of the AuPd nanostructure with a strong dependence on the composition.^57^ Theoretical calculations with a 201-atom model nanoparticle indicates a strong enthalpic driving force that favors Pd migration to the subsurface of the particle when the Au/Pd ratio is greater than 1, further suggesting some preference of Pd to be isolated from itself within Au.^58^ However, under an almost unitary Au/Pd ratio, as the approach in this work, the presence of Pd in some multinuclear ensembles is more probable assuming the nanoparticle size and shape.

With regard to colloidal stability, the ζ potential analyses revealed significant differences between the monometallic and bimetallic systems. Isolated Au NPs exhibited moderate stability (ζ potential = −19 mV), whereas Pd NPs showed an even lower value of −9 mV, suggesting a higher tendency of declining in their colloidal stability when compared to Au NPs. In contrast, the bimetallic AuPd nanoparticles displayed a ζ potential of −29 mV, close to the threshold generally associated with high colloidal stability (±30 mV), demonstrating the effectiveness of the adopted stabilization strategy. This enhanced stability is directly attributed to the combined action of *A. zerumbet* extract, which acted as a reducing/stabilizing agent in the initial step, and β-cyclodextrin, which provided an additional steric barrier, preventing agglomeration and ensuring good dispersion in aqueous media. In addition, we chose a slight excess of the CD/Au molar ratio (66.7) compared to the CD/Pd ratio (60) due to the possible increase in the stability of the initially prepared Au NP suspension. The DLS measurements revealed a Z-average diameter of 31 nm, which is a response of the intensity-weighted mean hydrodynamic size. This result is an approximation of the hydrodynamic diameter with uncertainties since it is based on the spherical model. A polydispersity index (PdI) of 0.506 further indicates that the system is not monodisperse in liquid suspension, likewise in the dry state.

## 4 Catalytic Performance of the AuPd NPs in the Reduction of Nitro Compounds

The reaction conditions were investigated using nitrobenzene (NB) as a model nitro compound (Figure 3) by varying the reaction time as well as the percentages of the catalyst and reducing agent.

**Figure 3:**
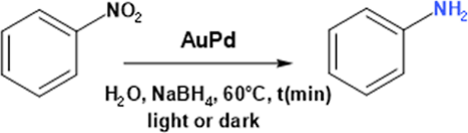
Reduction reaction of nitrobenzene to aniline.

First, control tests were performed under the following conditions: reaction time of 180 min, and 5 equiv of the reducing agent (NaBH_4_) using water or DMF as solvents but in the absence of the catalyst (Table 1, entries 1–4). The yields and selectivities were 8–19% and 55–94%, respectively, highlighting the negligible reduction of NB to aniline (AN) without the AuPd NPs (Figure S7).

**Table 1:** Catalytic Survey of the Reduction of Nitrobenzene to Aniline

entry	catalyst (mol %)	NaBH_4_/NB molar ratio	solvent	time (min)	condition	yield (%)	selectivity (%)
1	-	5	H_2_O	180	light	17.7	94.1
2	-	5	DMF	180	light	19.2	79.6
3	-	1	H_2_O	180	light	5.6	91.1
4	-	1	DMF	180	light	8.5	55.3
5	AuPd NPs [1 mol %]	5	H_2_O	90	light	98.4	98.4
5	AuPd NPs [1 mol %]	2.5	H_2_O	180	light	99.4	99.7
6	AuPd NPs [0,5 mol %]	5	H_2_O	90	light	77.2	89.9
7	AuPd NPs [0,5 mol %]	5	H_2_O	180	light	99.5	92.0
8	AuPd NPs [1 mol %]	2.5	H_2_O	90	light	93.2	99.9
9	AuPd NPs [1 mol %]	2.5	H_2_O	30	light	91.9	97.9
10	AuPd NPs [1 mol %]	1.25	H_2_O	30	light	53.0	69.3
11	AuPd NPs [1 mol %]	2.5	H_2_O	15	light	91.4	90.2
12	Pd NPs [1 mol %]	2.5	H_2_O	15	light	43.9	99.5
13	Au NPs [1 mol %]	2.5	H_2_O	30	light	1.4	41.3
14	AuPd NPs [1 mol %]	2.5	H_2_O	60	light	94.2	100
15	AuPd NPs [1 mol %]	2.5	H_2_O	15	dark	98.2	100
16	AuPd NPs [1 mol %]	10	H_2_O	15	light	94.8	95.7
17	AuPd NPs [1 mol %]	10	H_2_O	15	dark	95.3	88.8
18	AuPd NPs [1 mol %]	2.5	H_2_O	60	light	28.0	

Tests were then conducted with 1 mol % AuPd, yielding aniline with over 99% yield and selectivity (Table 1, entry 5) even with a reduction of reaction time from 180 to 90 min. Since it was inferred that the reaction kinetics are dependent on both the percentage of the AuPd catalyst and the amount of the reducing agent added, the AuPd percentage was reduced to 0.5 mol % in order to evaluate the catalytic response. As observed in Table 1, entry 6, the reaction yield reached 77% with 90% selectivity of aniline for a reaction time of 90 min. However, with a 2-fold increase in time (180 min), the aminated product was obtained with approximately 99% yield and 92% selectivity (Table 1, entry 7). On the other hand, keeping the AuPd content at 1 mol %, it was possible to achieve a yield of 90% (92% yield, 98% selectivity) in 30 min (Table 1, entry 9). Nevertheless, it should be noted that the quite low content of the reductant (1.25 equiv of NaBH_4_) significantly slowed the reaction (Table 1, entry 10) under the 30 min reaction time (53% yield). Therefore, the minimum loads of the catalyst and reducing agent required to achieve high aniline yields were 1 mol % of AuPd NPs and 2.5 equiv of NaBH_4_, respectively.

Under these conditions, the reaction was further optimized for AuPd NPs by reducing the reaction time to 15 min, and a high catalytic activity (91% yield and 90% selectivity) was indeed observed (Table 1, entry 11). In this case, a TOF of 393 h^–1^ was obtained. Additional reactions using only Au NPs or Pd NPs as catalysts demonstrated the synergy between the bimetallic nanoparticles. Specifically, in the reaction with Au NPs (Table 1, entry 13), almost no conversion of NB to AN occurred (1.5% yield). In contrast, the reaction catalyzed by Pd NPs yielded the aminated target product in approximately 44% yield (Table 1, entry 12). In addition, the condition described in Table 1, entry 11, was evaluated with a prolonged time (Table 1, entry 14). In this case, the observed yield confirmed the short reaction time and high selectivity.

To assess the reusability performance of the AuPd NPs, recycling was evaluated under the conditions described in Table 1, entry 11. Surprisingly, the abrupt decrease in the catalytic activity was also accompanied by some loss in selectivity (Table 1, entry 18). Therefore, we investigated possible morphological changes by TEM analyses after the catalytic reaction. According to Figure 5, a strong agglomeration of the bimetallic nanostructures occurred after the reaction. In addition, evidence of a certain gold enrichment of the AuPd particles revealed by EDS suggests the tendency of Pd leaching along with a possible preference of Pd to fill subsurface sites in bimetallic NPs.^58^

**Figure 4:**
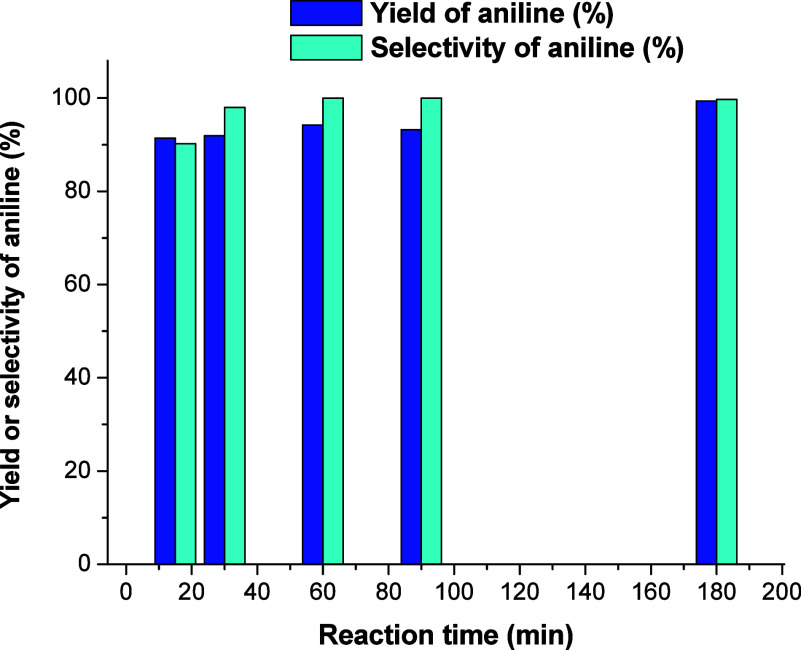
Selected catalytic data related to the yield or selectivity of aniline with reaction time.

**Figure 5:**
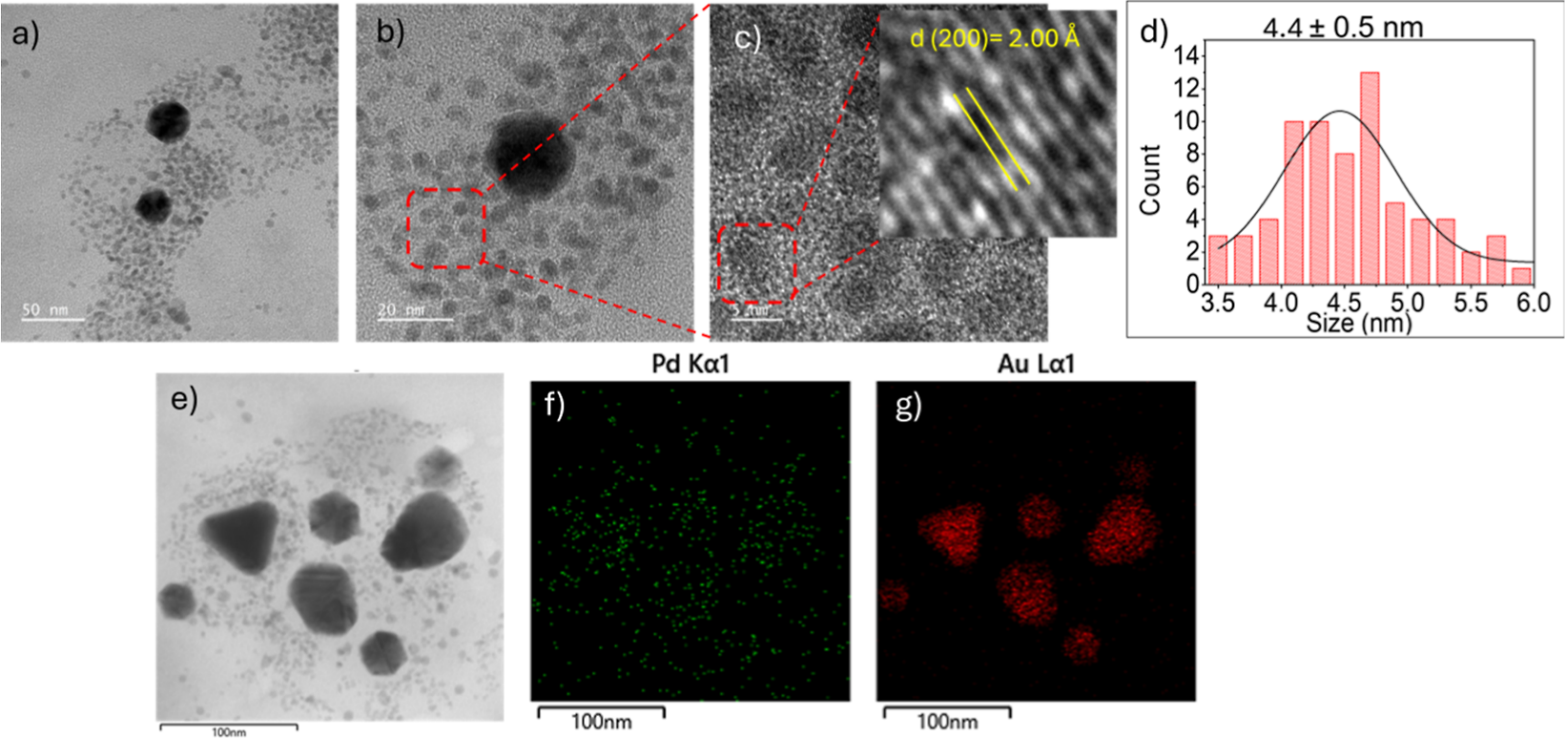
Bright-field representative TEM images of AuPd NPs after recycling (a, b); HRTEM of the recycled AuPd NPs showing the chosen area marked in the red dashed box for crystal d-spacing measurements (c); histogram with the particle size distribution of the recycled AuPd NPs (d); low-magnification TEM image of the recycled AuPd NPs (e); and EDS elemental mapping showing the Pd (f) and Au (g) distributions, represented as green and red dots, respectively.

The photoactivity of the AuPd NPs was previously attributed to the LSPR of Au NPs, acting as antennas and channeling light energy to the reaction medium containing Pd NPs.^33^ To embrace this hypothesis in the present context, catalytic tests of the model reaction were carried out under both light and dark conditions (Table 1, entries 11 and 15). The catalytic results showed that the reaction performed in the dark afforded a higher yield (98%, TOF = 393 h^–1^) compared to the reaction under illumination (91%, TOF = 366 h^–1^), both of them using 2.5 equiv of NaBH_4_. However, when the proportion of NaBH_4_ was increased, the reaction under light became more selective compared to the reaction in the dark (see Table 1, entries 16 and 17). In the first case, the effect may be associated with a slight deactivation of NaBH_4_ by reactive species formed during catalyst exposure to light. Upon absorption of a photon with energy corresponding to the LSPR of the Au NPs, electron oscillation may generate electron–hole pairs at the surface, which can subsequently react with hydroxyl ions to produce radicals. The formation of such radical species could lead to partial oxidation of NaBH_4_, thereby resulting in a lower catalytic performance of NaBH_4_ under light irradiation.^59^ However, when an excess of NaBH_4_ was employed in the reduction of nitroaromatics using Au NPs, no significant differences in yields were observed between reactions carried out under dark and illuminated conditions. In this second case, higher NaBH_4_ excesses may work in order to favor the reaction under light, since significant reductant species would be available in both dark and illuminated conditions, hence evidencing the occurrence of LSPR-based catalysis. Figures 4 and S12 illustrate some selected catalytic data considering different dependences of aniline yield(%) or selectivity(%).

In comparison with other studies reported in the literature focusing on nitroaromatic reduction reactions (Table 2), the AuPd NPs catalyst, which is the focus of this work, under the optimized and simplified reaction conditions, exhibited higher yield and TOF when compared with other catalysts (98% and a TOF of 393 h^–1^, Table 1, entry 15). Moreover, the other catalysts require the use of less environmentally friendly conditions, such as elevated temperatures and alcoholic solvents, which enhance the reducing potential of the reaction medium by acting as hydrogen donors to NB.

**Table 2:** Comparison of Catalytic Performances for Nitrobenzene Reduction to Aniline over Different Catalysts

substrate	year	catalyst	catalyst/Substrate molar ratio	NaBH_4_/Substrate molar ratio	temp. (°C)	solvent	time (min)	yield (%)	TOF (h^–1^)
nitrobenzene [this work]	-	AuPd NPs	0.01	2.5	60	H_2_O	15	98	393
4-nitrophenol [7]	2022	Au nanorods	42.3	66.7	30	H_2_O	24	>90	-
nitrobenzene [10]	2022	CuFeS_2_ NCs	5.5	16	54	H_2_O	240	84	22
nitrobenzene [11]	2023	TiO_2_ P25	-	-	25	MeOH	180	>80	-
nitrobenzene [12]	2013	Ce_2_S_3_	400	30.4	30	iPrOH	300	34	
nitrobenzene [14]	2022	Bi_2_MoO_6_	325	8	28	iPrOH	600	86	0
4-nitrophenol [3]	2020	Au_©-CD NP	-	44	25	H_2_O	8	-	43
4-nitrophenol^60^	2023	Ag/PSZN-5	-	1000	25	H_2_O	3.3	99,2	786.61
4-nitrophenol^61^	2025	CNTs@CeO2–Au/SnO_2_	-	378.79	25	H_2_O	2.8	100	1.744
 ^62^	2019	Au@g-C_3_N_4_	0.5	40	25	H_2_O	10	99	12
 ^63^	2024	Au–Pd	-	-	15	EtOH	240	-	166

The AuPd nanocomposite was also tested in the reduction of other nitroaromatics, such as 3-nitroaniline and *p*-nitrophenol, under the optimal reaction conditions determined from the nitrobenzene-to-aniline reduction (Figure 6). The aminated products were obtained in high yields (4-aminophenol at 70% and 1,3-diaminobenzene at 95%), demonstrating the versatility of the catalyst.

**Figure 6:**
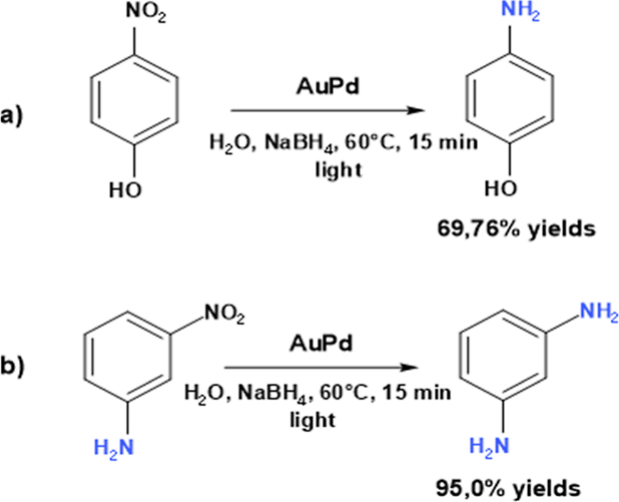
Reduction reaction of 4-nitrophenol (a) and 1,3-nitroaniline (b).

Figure 7 shows a proposed simplified pathway for the reduction of nitrobenzene. From the results, it is possible to consider a catalytic synergy between the Au and Pd sites. Even though Au sites do not directly contribute to the reduction of NB, it is possible that borohydride anions could be preferentially adsorbed on gold sites and a hydrolytic cleavage of the reductant can form active hydrogen species.^46^ In addition, considering the associate constant between nitrobenzene and β-cyclodextrin adsorbed onto the bimetallic alloy sites, the possible formation of a supramolecular complex cannot be ruled out. Finally, the suggested evidence for the formation of reactive species, upon light or dark on the alloy surface, allows for the successive hydrogen transfer processes involved in the formation of aniline. Significantly, isolated Pd sites do not efficiently catalyze the reduction reaction.^64–66^

**Figure 7:**
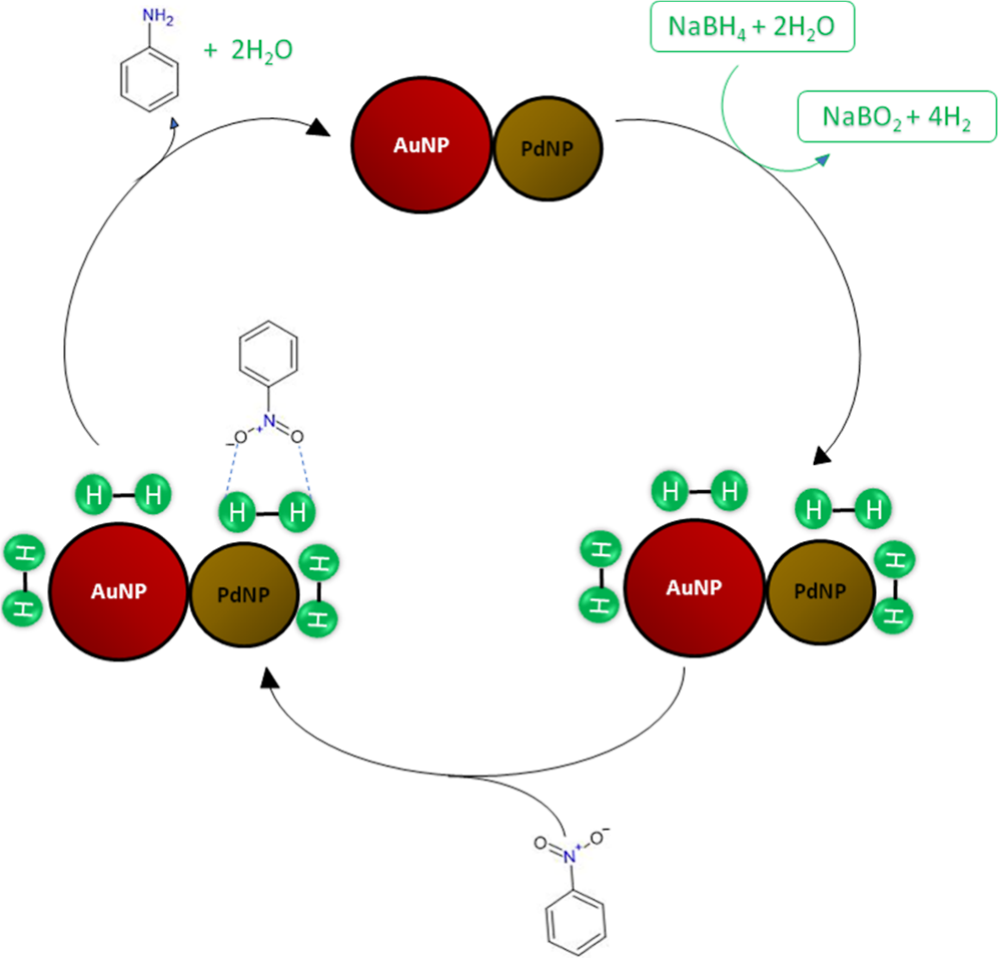
Proposed catalytic cycle for the nitrobenzene reduction reaction in the presence of AuPd NPs.

Following the expected thermodynamic tendency observed for the catalytic hydrogenation of nitrobenzene derivatives by supported Au and Pd catalysts, we believe that all the hydrogenation reactions were exothermal and thermodynamically favorable, but with gold sites presenting higher affinity toward the adsorption of the reductant precursor.^67^

## 5 Conclusion

In summary, the eco-friendly synthesis route proved to be highly effective, as the *A. zerumbet* extract successfully acted as both reducing and stabilizing agents for Au nanoparticles, enabling the formation of AuPd NPs with conceivable ligand alloy effects, as suggested in the morphology observed along with the catalytic performance of the nitroarenes reduction. XRD, HRTEM, DLS, and UV–vis analyses provided evidence for the structural and optical properties of the NPs. TEM images revealed that AuPd nanoparticles displayed a bimodal size distribution with a crystalline nature. Zeta potential measurements highlighted that AuPd nanoparticles showed improved electrostatic stabilization. The catalytic results toward the reduction of nitrobenzene clearly demonstrated the synergistic effect between Au and Pd, suggesting the formation of a nanoalloy. While Au nanoparticles alone exhibited negligible activity and Pd nanoparticles achieved only moderate conversion, the AuPd nanocomposite delivered nearly quantitative yields of aniline within very short reaction times. Under optimized conditions, the catalyst promoted nitrobenzene reduction with yields above 98% in only 15 min, corresponding to an outstanding TOF of 393 h^–1^. Such performance surpasses that of other catalytic systems reported in the literature, which often require harsher conditions, higher catalyst loadings, or the use of organic solvents.

Finally, the catalyst also demonstrated versatility by effectively reducing other nitroaromatic substrates, including 3-nitroaniline and 4-nitrophenol, yielding the corresponding aminated products with high selectivity and good-to-excellent yields. Taken together, these findings establish the AuPd NPs synthesized with *A. zerumbet* extract as a robust, efficient, and sustainable catalytic system for the nitroaromatic reduction.

## Supplementary Material



## Notes

1. Chemical structures identified in the *A. zerumbet* extract; 2. qualitative characterization of polyphenols; 3. Folin–Ciocalteu method; 4. HPLC–DAD analyses of *A. zerumbet* extract at different wavelengths; 5. cyclic voltammetry; 6. UV–vis spectrum of the extract; 7. Fourier transform infrared (FTIR) spectra of AuPd NPs; 8. SEM–EDS images of AuPd NPs; 9. ^1^H NMR spectroscopy of reaction products; 10. determination of yields; 11. XPS data; 12. zeta potential analyses; and 13. selectivity of aniline under light and dark conditions (PDF)
