# Supplementary material for: Eco-Friendly Synthesis of AuPd Bimetallic Nanoparticles Using Alpinia zerumbet for Efficient Reduction of Nitro Compounds
Source: ACS Omega. 2026 Mar 18;11(12):18704–14. doi: 10.1021/acsomega.5c09011 (PMC13044683; doi:10.1021/acsomega.5c09011)
Supplement: Supplementary file 1 [file ao5c09011_si_001.pdf]

Supplementary Information

Eco-Friendly Synthesis of AuPd Bimetallic Nanoparticles Using *Alpinia zerumbet* for Efficient Reduction of Nitro Compounds

Ana Paula Nazar de Souza[a], Gabriel Francisco Souza da Silva[b], Evelyn C. S. Santos[c], Jefferson S. de Gois[a], Cesar Augusto D. Mendoza[d], Suellen Dayenn Tozetti de Barros[e] , Marcelo E. H. Maia da Costa[e], Marcelo Augusto Vieira de Souza[a], Luiz Fernando B. Malta[b], Nakédia M. F. Carvalho[a], Jaqueline D. Senra[a]*

[a]Rio de Janeiro State University, Chemistry Institute, Rua São Francisco Xavier 524 – Maracanã, Rio de Janeiro, RJ, Brazil, 20550-900.
[b] Rio de Janeiro Federal University, Av. Athos da Silveira Ramos 149, Bloco A, Cidade Universitária,Rio de Janeiro, RJ, Brasil, 21941-909.
[c] Centro Brasileiro de Pesquisas Físicas, Urca, 22290-180, Rio de Janeiro, RJ, Brazil.
[d] Rio de Janeiro State University, Department of Electric Engineering, Rua São Francisco Xavier 524 – Maracanã, Rio de Janeiro, RJ, Brazil, 20550-900.
[e] Department of Physics, Pontifical Catholic University of Rio de Janeiro, Rua Marques de São Vicente, 22451-900, Rio de Janeiro, Brazil.

E-mail: jaqueline.senra@uerj.br

Table of Contents

1. Chemical structures identified in the *Alpinia zerumbet* extract........................S02
2. Qualitative characterization of polyphenols..S03
3. Folin−Ciocalteu method...S04
4. HPLC- DAD analyses of *Alpinia zerumbet* extract at different wavelength.......S05
5. Cyclic voltammetry of *Alpinia zerumbet* extract..S09
6. UV – vis spectrum of *Alpinia zerumbet* extract S10
7. Fourier-transform infrared spectra of AuPd NPs..S11
8. SEM-EDS Images of AuPd NPs..S12
9. XPS data..S16
10. [1] H NMR Spectroscopy S18
11. Determination of yields..S19
12. Zeta Potential analyses...S20
13. Selectivity of aniline in light and dark..S21
14. References...S22

1. Chemical structures identified in the *Alpinia zerumbet* extract [1]

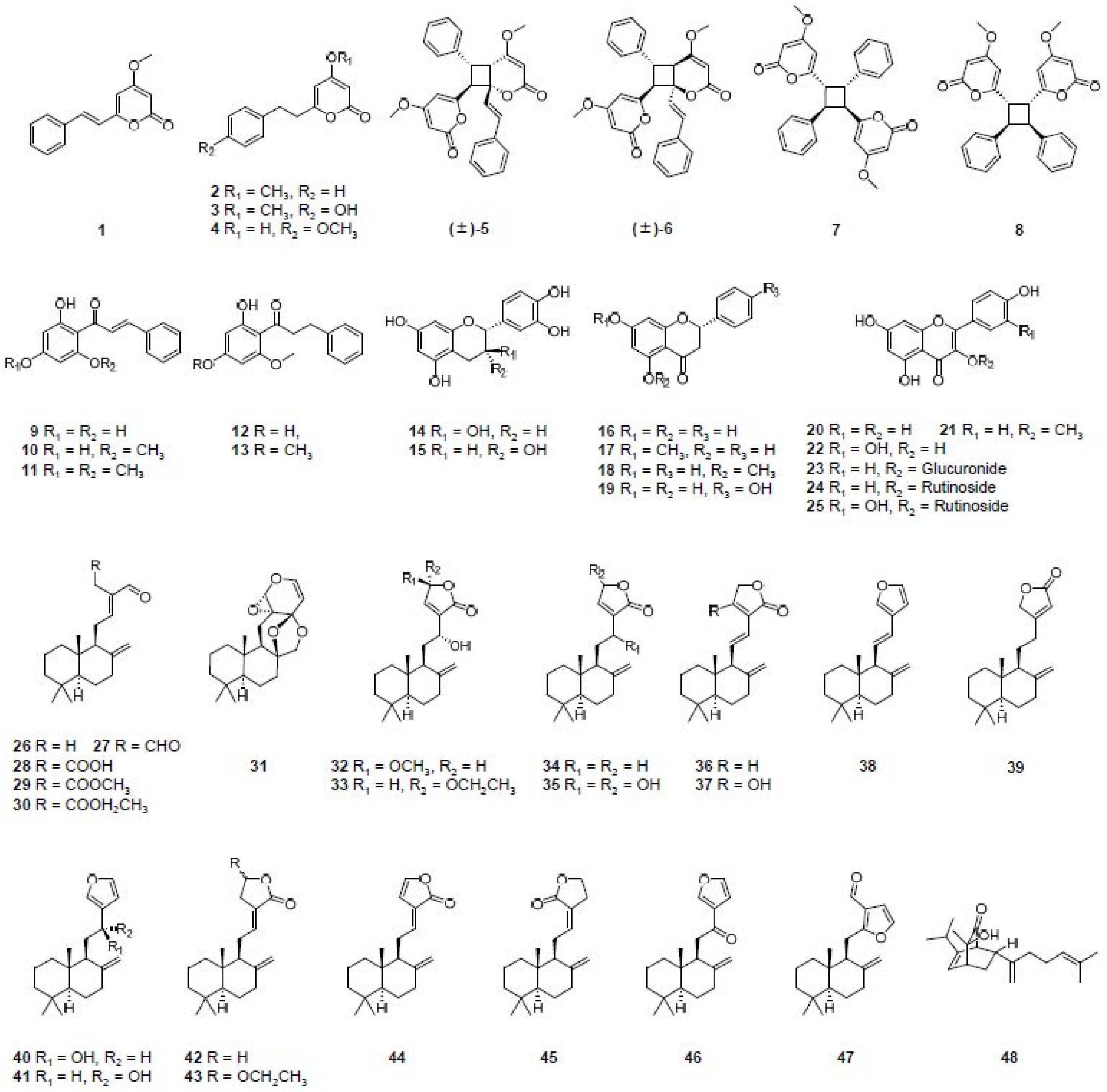

Figure S1. Structures of the kavalactones, chalcones, flavonoids, diterpenoids, and sesquiterpenoids isolated from *A. zerumbet*[1].

2. Qualitative characterization of polyphenols

For the qualitative determination of phenolic compounds, 2 mL of the hydroalcoholic extract of *Alpinia zerumbet* (Colônia) were treated with five drops of 5% $FeCl_3 \cdot 6H_2O$. For the detection of tannins, 2 mL of the extract were similarly treated with five drops of 10% $FeCl_3 \cdot 6H_2O$. A positive reaction was indicated by either a color change or the formation of a precipitate[2]. The presence of flavonoids was evaluated by adding approximately 0.5 cm of magnesium ribbon and 2 mL of concentrated hydrochloric acid to 2 mL of the extract. Effervescence was observed during the reaction, and the appearance of a brown to red coloration confirmed the presence of flavonoids[2].

Coumarins were assessed by mixing 2 mL of the extract with five drops of 10% NaCl, where the development of a yellow coloration indicated a positive result. Terpenoids were detected by combining 2 mL of the extract with 1 mL of chloroform and three drops of concentrated sulfuric acid, with the appearance of a gray coloration confirming their presence. Quinones were identified by adding 5 mL of hydrochloric acid to 2 mL of the extract, with the formation of a yellow precipitate serving as evidence of their occurrence[3-4]. For the detection of alkaloids, 2 mL of the alkalinized extract were treated with fifteen drops of 1% sodium hydroxide, 2 mL of distilled water, and 2 mL of chloroform. After discarding the aqueous phase, fifteen drops of 1% hydrochloric acid were added to the organic fraction, followed by 2 mL of distilled water. The chloroform fraction was discarded, and the tests were conducted using the acidic aqueous phase. Three drops of Dragendorff's reagent were then added, with the formation of insoluble, flocculent precipitates confirming the presence of alkaloids[5].

Table S1. Qualitative analysis of hydroalcoholic extract of *Alpinia zerumbet*.

Phenolic compounds	Alkaloids	Coumarins	Tannins	Quinones	Terpenoids	Flavonoids
+	-	-	+	-	-	+

3. Folin−Ciocalteu method[6]

An aliquot of 0.5 mL of the extract was diluted to 10 mL in volumetric flask. 1 mL of this solution was transferred to a test tube, in which 5 mL of the Folin-Ciocalteu reagent was added, diluted in distilled water 1:10 (v/v) and 4 mL of calcium carbonate 20% (w/v). The mixture was slightly hand-shaken and incubated at 50 °C for 8 min. Then, at room temperature, the absorbance was measured in a UV–Vis spectrophotometer with a diode-array Agilent 8453 (USA) at 760 nm. Total phenolic content was determined by comparison with a standard curve of gallic acid (5, 10, 15, 20, 25, and 30 mg L^{-1}) and expressed in terms mg EGA g^{-1} of the hydroalcoholic extract of *Alpinia zerumbet*.

$$weight_{\ gallic\ acid\ in\ solution} = \frac{mg\ acid}{1000ml\ of\ solution}\ x\ final\ Volume\ (ml) \quad \textbf{Eq. S1}$$

$$Concentration_{\frac{w}{w}} = \frac{mass\ (mg)_{gallic\ acid\ in\ solution}}{mass\ (g) of\ Alpinia\ zerumbet} \quad \textbf{Eq. S2}$$

4. HPLC- DAD analyses of *Alpinia zerumbet* extract at different wavelengths.

The concentration of the compounds was determined by HPLC-DAD. Peak area measurements were performed on an Agilent 1260 Infinity instrument with an autosampler, gradient elution, diode array detector (DAD) (λ = 280, 320, and 360 nm), and a Pursuit 5 C18 column (250 x 4.6 mm i.d., 5 µm particle size). The flow rate was 1.0 mL min^{-1} with an injection volume of 20 µL and a column temperature of 25°C. Individual standards were prepared in methanol. The 10 mg L^{-1} mixed standard was prepared in the mobile phase (initial gradient) (95% solvent A + 5% solvent B) and used to prepare the analytical curve.

Table S2. Retention times (rt) (min)

λ (nm)	Gallic	Protocatechoic	Vanillic	Syringic	Trans-cinnamic	Caffeic	Coumaric	Rutin	Quercetin
**280**	5.115	8.813	16.328	17.732	42.231				
**320**						16.87	23.369		
**360**								27.346	41.46

Table S3. Concentration of polyphenols in mg L^{-1}.

Gallic	Protocateic	Vanillic	Syringic	Trans-cinnamic	Caffeic	Coumaric	Rutin	Quercetin
1.8	0.72	0.17	0.43	1.5	2.8	0.52	5.8	< 0.02

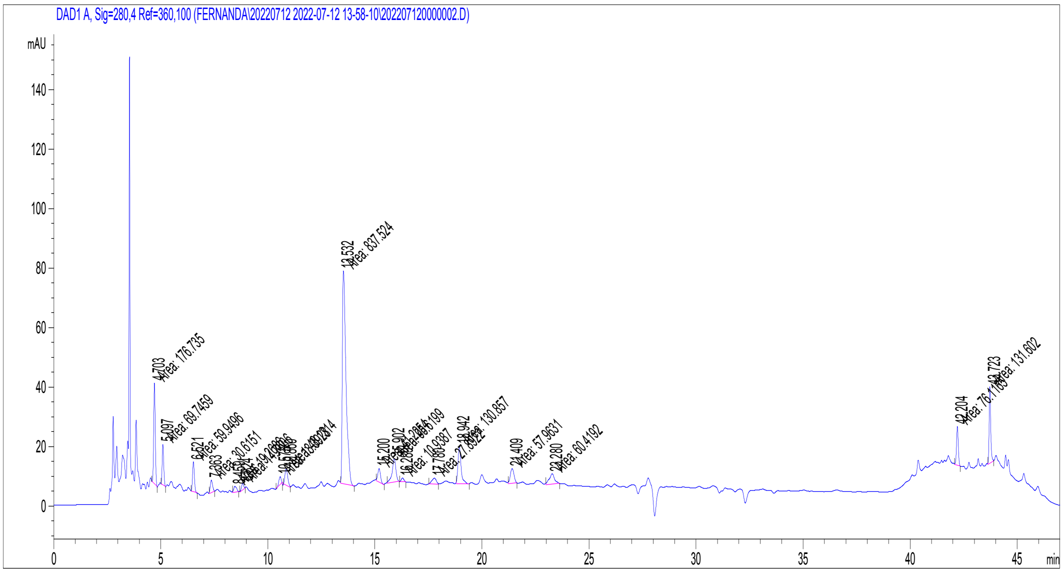

#	Time	Area	Height	Width	Area%	Symmetry
1	4.703	176.7	34	0.0868	9.264	0.902
2	5.097	69.7	13.7	0.0848	3.656	0.814
3	6.521	59.9	10.1	0.0987	3.142	0.871
4	7.363	30.6	4.2	0.1225	1.605	0.851
5	8.459	19.3	2	0.1596	1.010	0.696
6	8.824	14.8	2.1	0.1152	0.774	1.255
7	10.573	18.4	2.7	0.1142	0.964	1.443
8	10.86	48.1	5.4	0.1481	2.523	0.728
9	13.532	837.5	71.6	0.195	43.901	0.435
10	15.2	41.2	4.6	0.1508	2.161	0.866
11	15.902	95.6	7.9	0.2008	5.012	1.231
12	16.289	10.9	1.3	0.1432	0.573	0.897
13	17.78	27.9	2	0.237	1.462	1.258
14	18.942	130.9	12.7	0.1723	6.859	0.73
15	21.409	58	4.9	0.1955	3.038	0.988
16	23.28	60.4	3.5	0.2845	3.167	1.252
17	42.204	76.1	13	0.0972	3.990	0.917
18	43.723	131.6	25.8	0.0851	6.898	0.817

Figure S2a. HPLC- DAD analysis of *Alpinia zerumbet* extract at $\lambda = 280$ nm

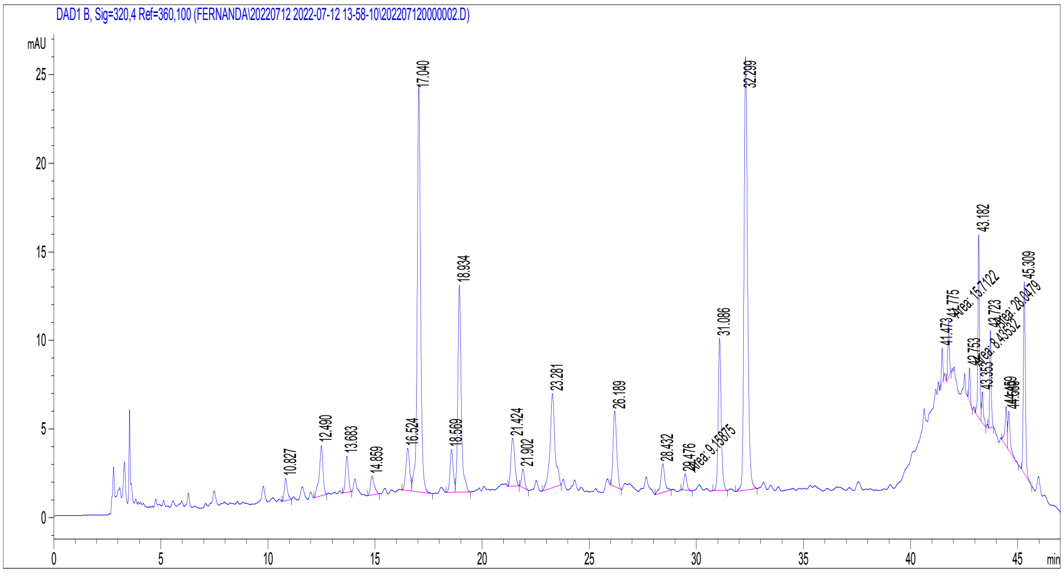

#	Time	Area	Height	Width	Area%	Symmetry
1	10.827	11.9	1.2	0.1463	0.897	0.741
2	12.49	34.7	2.8	0.1807	2.624	1.354
3	13.683	20.2	2.1	0.1505	1.525	0.897
4	14.859	13	1.1	0.186	0.981	0.65
5	16.524	29.7	2.4	0.186	2.243	0.965
6	17.04	257.4	23	0.1686	19.457	0.884
7	18.569	25.7	2.4	0.1611	1.939	0.873
8	18.934	130.1	11.7	0.1681	9.835	0.758
9	21.424	33.4	2.7	0.1921	2.521	0.807
10	21.902	10.1	1	0.1531	0.764	0.838
11	23.281	84.4	5.3	0.2295	6.379	0.962
12	26.189	49.8	4.3	0.1819	3.760	0.811
13	28.432	21.6	1.6	0.2007	1.635	1.018
14	29.476	9.2	9.3E-1	0.1646	0.692	0.786
15	31.086	84.2	8.6	0.1482	6.363	0.84
16	32.299	279.4	24.4	0.1735	21.118	0.778
17	41.473	8.8	1.9	0.0707	0.663	1.121
18	41.775	15.7	3.2	0.081	1.187	0.995
19	42.753	8.4	1.9	0.0755	0.638	0.813
20	43.182	54.2	10.4	0.079	4.096	0.912
21	43.353	9.7	1.7	0.0817	0.736	0.651
22	43.723	28	5.5	0.0845	2.120	0.793
23	44.459	15.7	2.2	0.1023	1.186	1.311
24	44.589	15.8	2.2	0.1053	1.191	0.611
25	45.309	72.1	10.8	0.1016	5.450	0.809

Figure S2b. HPLC- DAD analysis of *Alpinia zerumbet* extract at λ = 320 nm.

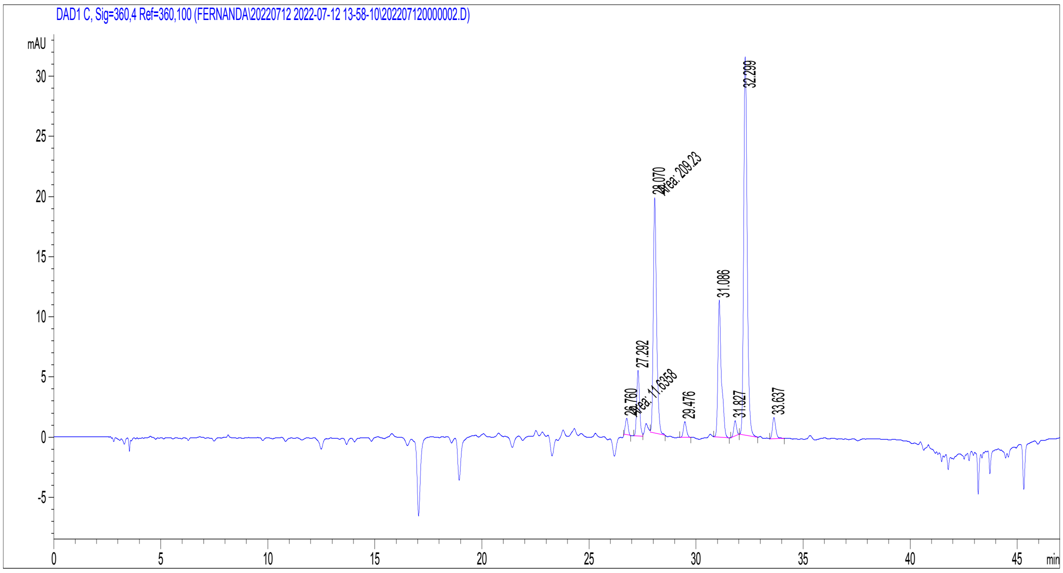

#	Time	Area	Height	Width	Area%	Symmetry
1	26.76	11.6	1.4	0.1424	1.461	0.925
2	27.292	51.1	5.5	0.1454	6.418	0.847
3	28.07	209.2	19.6	0.1779	26.265	0.749
4	29.476	13.3	1.3	0.1569	1.666	0.833
5	31.086	129.5	11.4	0.1648	16.250	0.597
6	31.827	11.5	1.3	0.1423	1.444	0.997
7	32.299	351.3	31.4	0.1704	44.099	0.768
8	33.637	19.1	1.8	0.1669	2.397	0.752

Figure S2c. HPLC- DAD analysis of *Alpinia zerumbet* extract at $\lambda = 360$ nm.

5. Cyclic voltammetry of *Alpinia zerumbet* extract

The hydroalcoholic extract was characterized by cyclic voltammetry in phosphate buffer (pH 6.7), using an Autolab PGSTAT302N potentiostat/galvanostat, Metrohm, Switzerland. A three-electrode system was used as the electrolytic cell, using a freshly polished 3.0 mm glassy carbon as the working electrode (Metrohm), Pt rod as the counter electrode (Metrohm) and Ag|AgCl (KCl 3.0 mol L^{-1}) as the reference electrode, from 0 to 1 V at a scan rate of 100 mV s^{-1}. To convert the potential from the Ag|AgCl to the normal hydrogen electrode (NHE), +0.210 V was added to the measured potential.

E_{pa} = +290 mV vs Ag/AgCl = +500 mV vs NHE

E_{pc} = +200 mV vs Ag/AgCl = +410 mV vs NHE

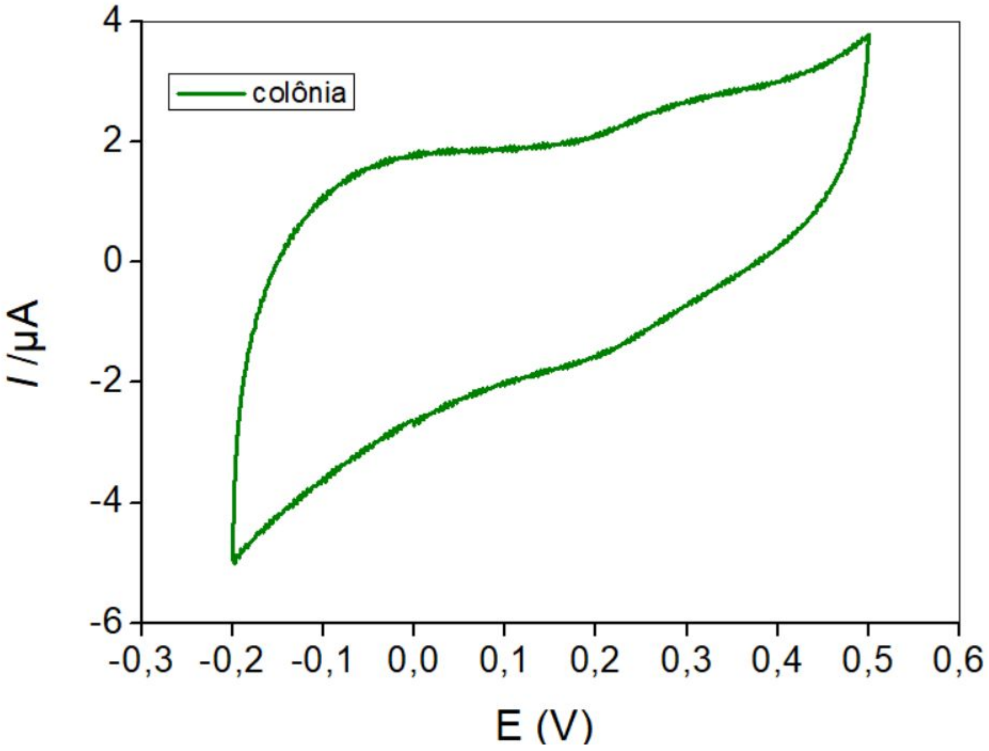

Figure S3. Cyclic voltammetry of *Alpinia zerumbet* extract.

6. UV – vis spectrum of *Alpinia zerumbet* extract.

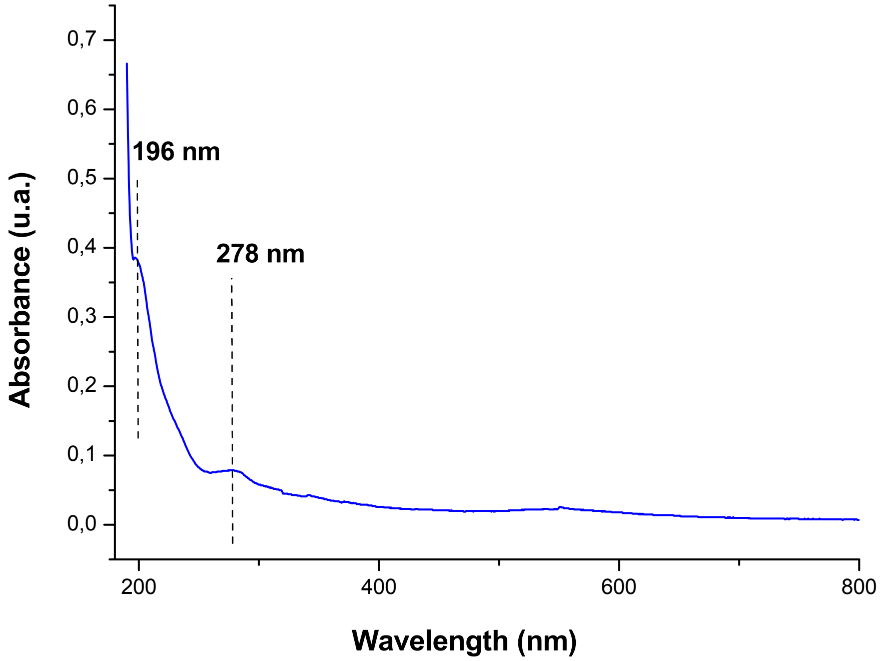

Figure S4. UV – vis spectrum of *Alpinia zerumbet* extract.

7. Fourier-transform infrared spectra of AuPd NPs

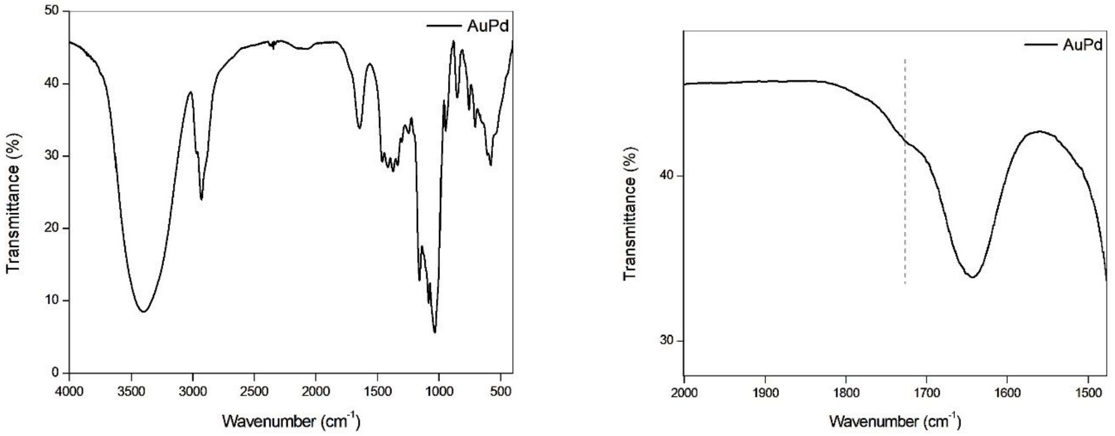

Figure S5. Fourier-transform infrared spectra of AuPd NPs.

8. SEM-EDS analyses of AuPd NPs

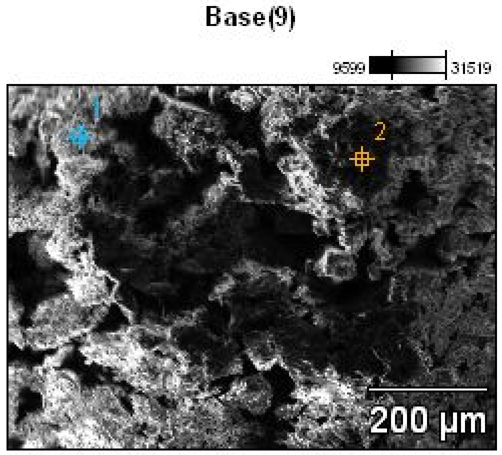

Figure S6a. SEM image of AuPd NPs (Accelerating Voltage: 15.0 kV, Magnification: 140)

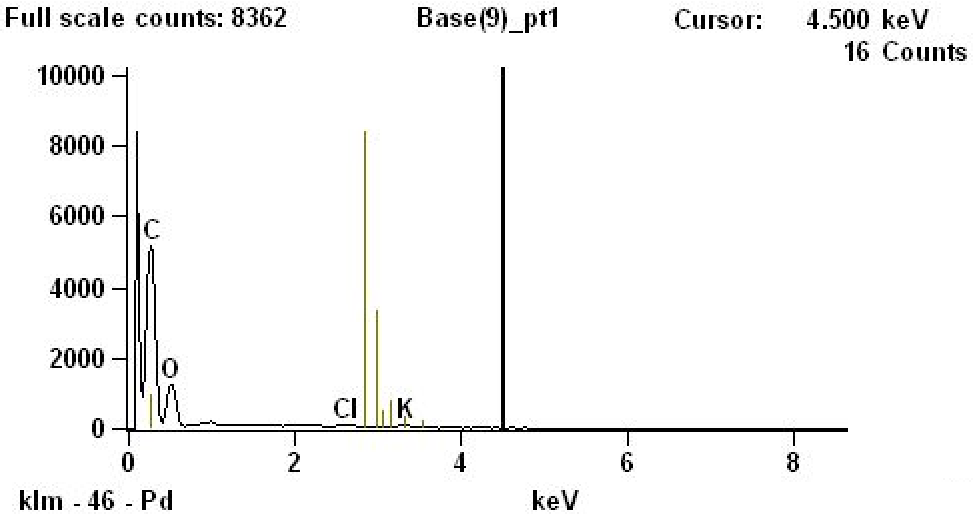

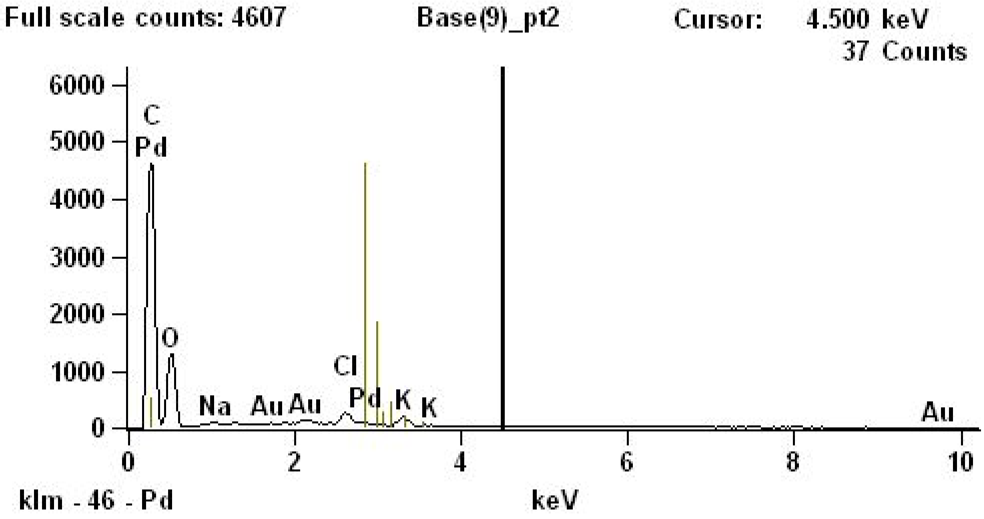

Net Counts

	C-K	*O-K*	*Na-K*	*Cl-K*	*K-K*	*Pd-L*	*Au-M*
Base(9)_pt1-No Data.							
Base(9)_pt2	33783	9995	359	2605	2199	309	1034

Weight %

	C-K	*O-K*	*Na-K*	*Cl-K*	*K-K*	*Pd-L*	*Au-M*
Base(9)_pt1-No Data.							
Base(9)_pt2	47.92	44.94	0.49	2.24	2.36	0.48	1.56

Atom %

	C-K	*O-K*	*Na-K*	*Cl-K*	*K-K*	*Pd-L*	*Au-M*
Base(9)_pt1-No Data.							
Base(9)_pt2	57.36	40.38	0.31	0.91	0.87	0.07	0.11

Figure S6b. EDS spectra and molar ratio percent of C, O, Na, Cl, K, Pd and Au.

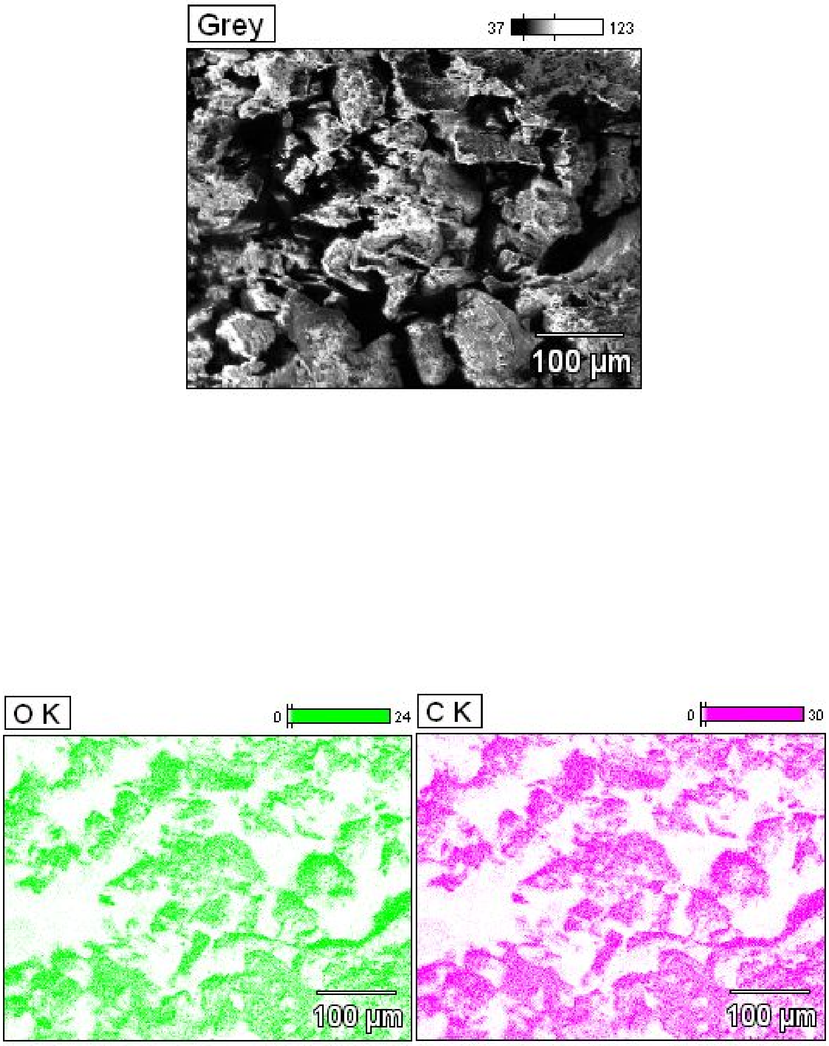

Figure S6c. SEM image (SE) of AuPd NPs and EDS element mapping image (O and C distribution).

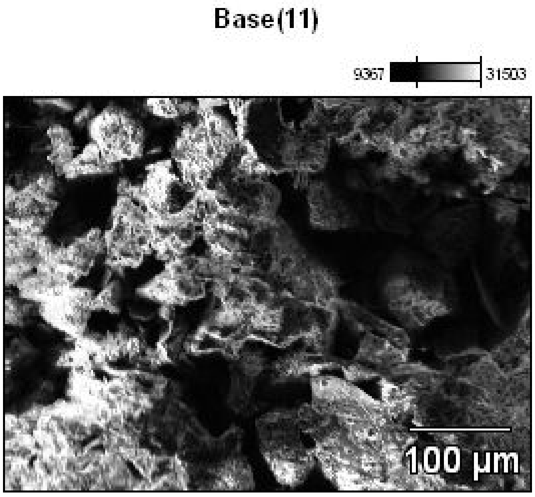

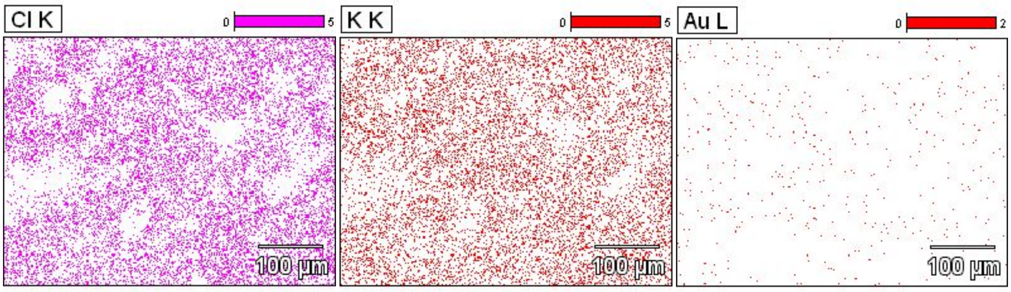

Figure S6d. SEM image (SE) of AuPd NPs and EDS element mapping image (Cl, K and Au distribution).

9. XPS data

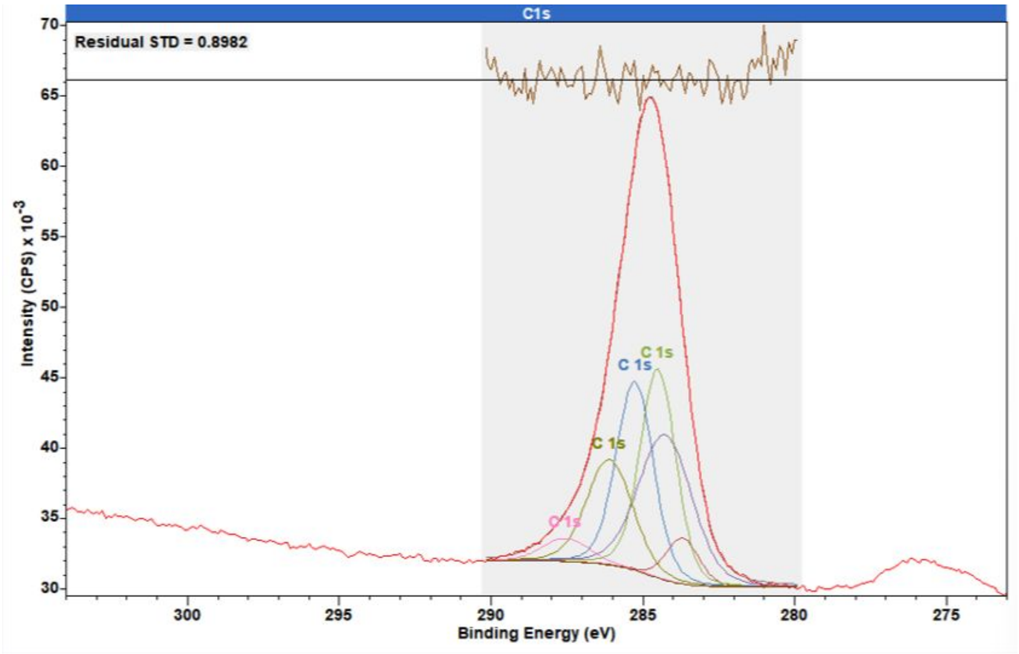

Figure S7a. High-resolution XPS spectrum of C 1s.

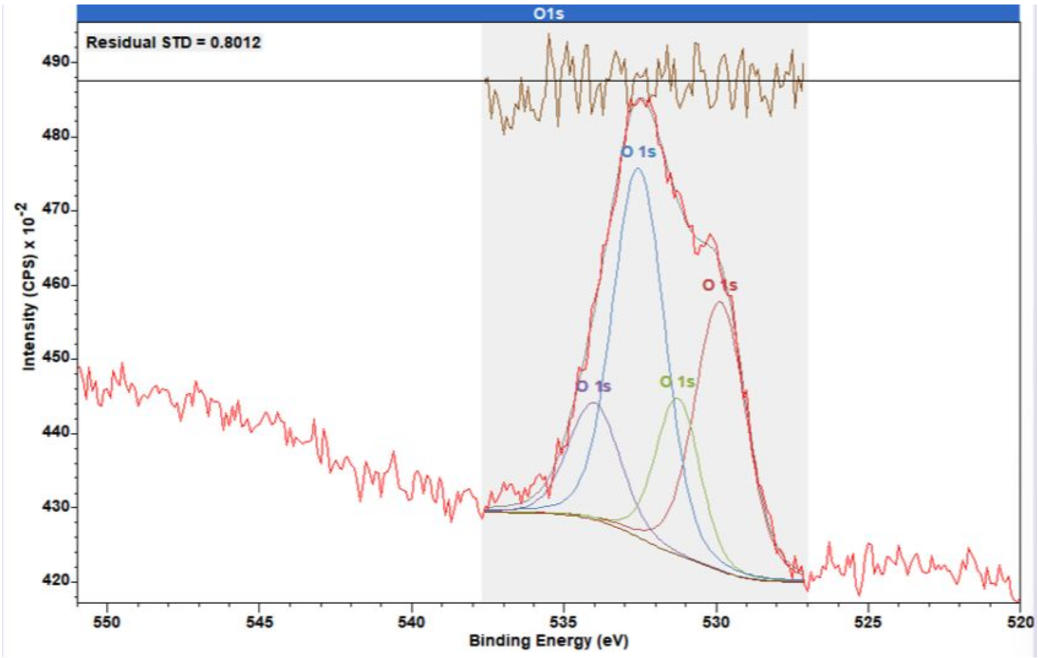

Figure S7b. High-resolution XPS spectrum of O 1s.

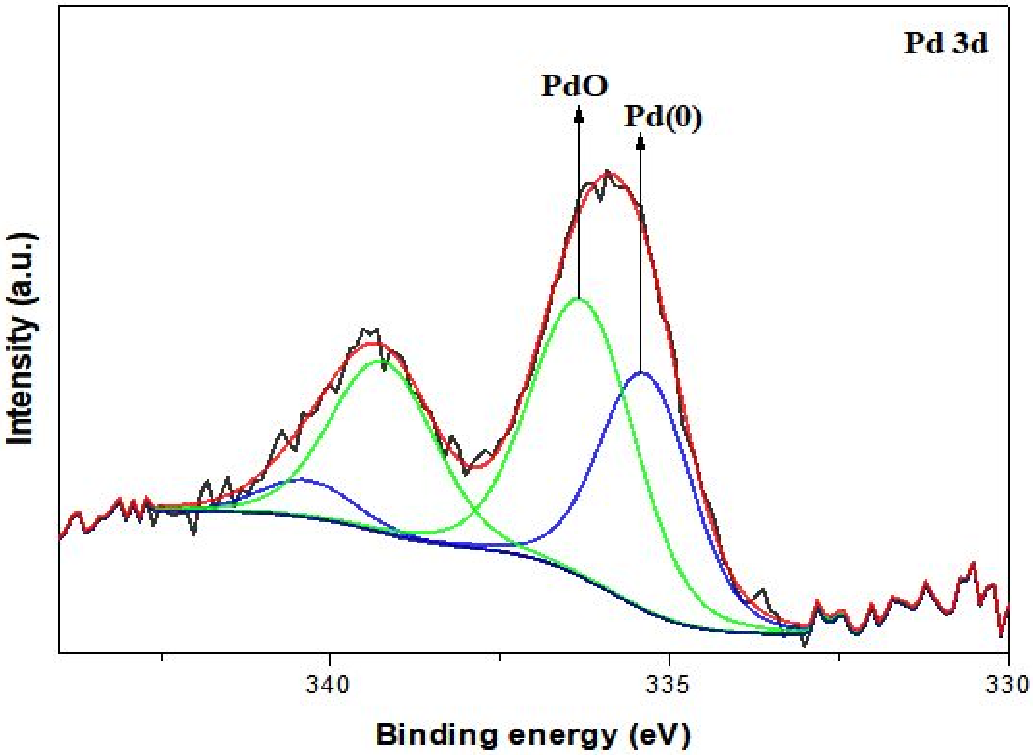

Figure S7c. High-resolution XPS spectrum of Pd 3d.

10. [1] H NMR Spectroscopy

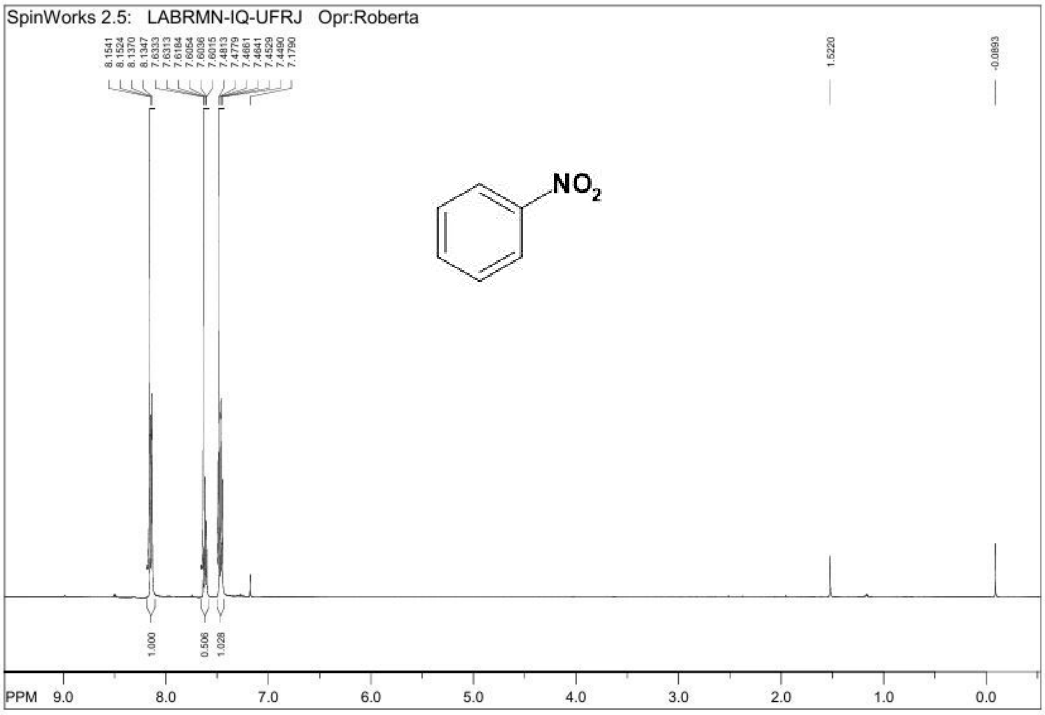

Figure S8a. ^{1}H NMR of nitrobenzene ($CDCl_3$).

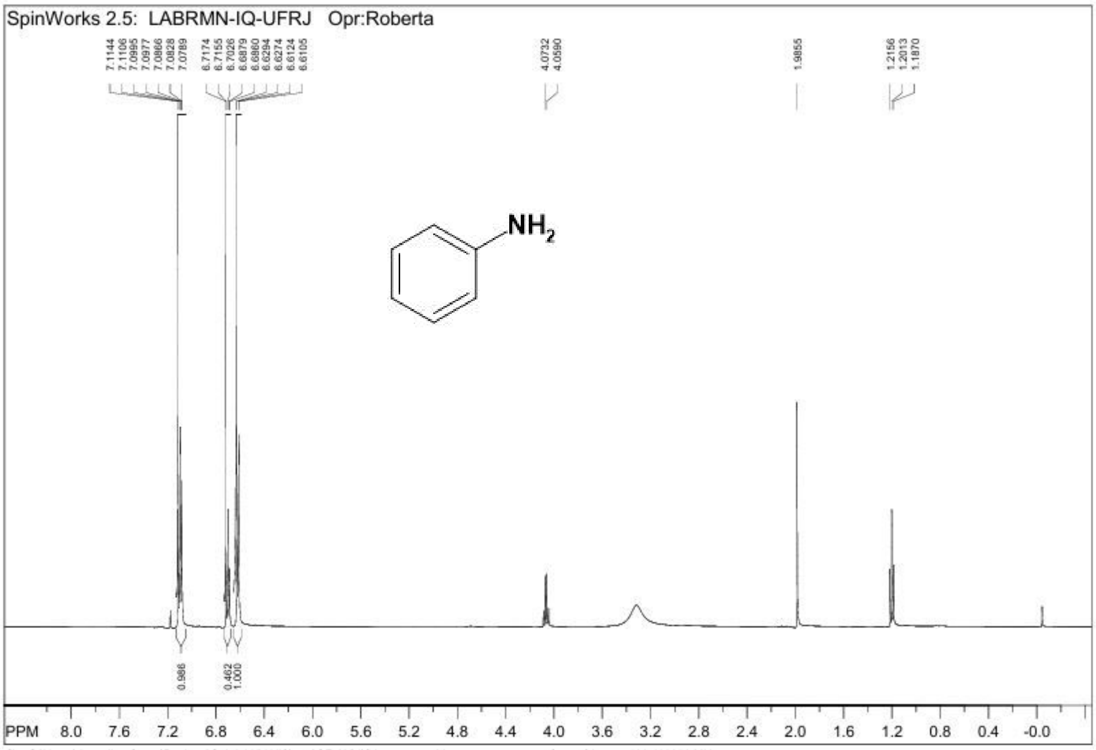

Figure S8b. ^{1}H NMR of Aniline ($CDCl_3$).

11. Determination of yields

After the reaction, the product was separated by liquid-liquid extraction with ethyl acetate and then dried over anhydrous Na_2SO_4. The product was analyzed by gas chromatography (GC) (Agilent 7890B), equipped with a flame ionization detector (FID). The products were identified by comparison of retention times with chemical standards.

The equations used to calculate conversion, yield, and selectivity are presented below.

$$\text{Yield (\%)} = \frac{Peak\ area\ of\ desired\ product}{Total\ peak\ area} \text{ x } 100 \qquad \textbf{Eq. S3}$$

$$\text{Selectivity (\%)} = \frac{Peak\ area\ of\ desired\ product}{Peak\ area\ of\ products} \text{ x } 100 \qquad \textbf{Eq. S4}$$

Turnover frequency (TOF) was calculated according to the following equation:

$$\text{TOF } (h^{-1}) = \frac{Mols\ of\ desired\ product}{Mols\ of\ catalyst\ x\ reaction\ time} \qquad \textbf{Eq. S5}$$

The use of peak areas in the yield calculations by GC-FID was made possible through the previous determination of the response factor of products.

12. Zeta potential analyses

Table S4. ζ potential analyses of Au NPs, Pd NPs and AuPd NPs.

Sample	Measurement	ζ-Potential (mV)
	1	−18.3
**Au NPs**	2	−20.1
	3	−19.6
	**Mean ± SD**	**−19.3 ± 0.9**
	1	−8.7
**Pd NPs**	2	−8.8
	3	−9.1
	**Mean ± SD**	**−8.9 ± 0.2**
	1	−27.6
**AuPd NPs**	2	−29.2
	3	−30.9
	**Mean ± SD**	**−29.2 ± 1.7**

13. Selectivity of aniline in light and dark

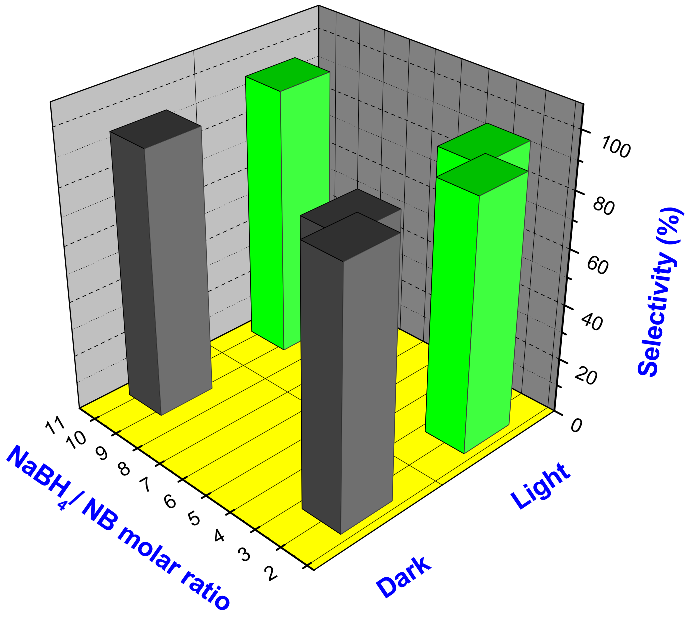

Figure S9. Comparison of the aniline selectivity and $NaBH_4$/ NB molar ratio in the light and in the dark. (NB = nitrobenzene).

14. References

(1) Nishidono, Y.; Tanaka, K. Phytochemicals of *Alpinia zerumbet*: A Review. *Molecules* **2024**, *29*, 2845. https://doi.org/10.3390/molecules29122845.

(2) Gul, R.; Jan, S. U.; Faridullah; Sherani, S.; Jahan, N. Preliminary Phytochemical Screening, Quantitative Analysis of Alkaloids, and Antioxidant Activity of Crude Plant Extracts from *Ephedra intermedia* Indigenous to Balochistan. *Sci. World J.* **2017**, 5873648. https://doi.org/10.1155/2017/5873648.

(3) Ganeshan, P.; Joghee, S.; Vincent, A. Ecofriendly Biosynthesis of Zinc Oxide and Magnesium Oxide Particles from Medicinal Plant *Pisonia grandis* R.Br. Leaf Extract and Their Antimicrobial Activity. *BioNanoScience* **2019**, *9*, 141–154. https://doi.org/10.1007/s12668-018-0573-9 .

(4) Costa, T. A. C. *Perfil Fitoquímico de Materiais Biológicos Usados em Dessalinizador Caseiro de Água Salobra*. Master's Thesis, Universidade Federal da Bahia, Salvador, Brazil, 2011. https://repositorio.ufba.br/handle/ri/10676.

(5) Silva, N. L. A.; Miranda, F. A. A.; Conceição, G. M. Triagem Fitoquímica de Plantas do Cerrado da Área de Proteção Ambiental Municipal do Inhamum, Caxias, Maranhão. *Scientia Plena* **2010**, *6*, 1–6. https://scientiaplena.emnuvens.com.br/sp/article/view/22.

(6) Singleton, V. L.; Rossi, J. A., Jr. Colorimetry of Total Phenolics with Phosphomolybdic–Phosphotungstic Acid Reagents. *Am. J. Enol. Vitic.* **1965**, *16*, 144–158. https://www.ajevonline.org/content/16/3/144.
